# Structured Aluminum Phosphonate Metal–Organic Framework for Efficient Hydrogen Purification: Reversible HCl Adsorption and Water-Triggered Regeneration

**DOI:** 10.1021/acs.chemmater.5c03397

**Published:** 2026-04-10

**Authors:** Ravi Sharma, Ajay Padunnappattu, Max Phil Van der Stricht, Norbert Stock, Joeri F. M. Denayer

**Affiliations:** a Chemical Engineering Department, 70493 Vrije Universiteit Brussel, Brussel B-1050, Belgium; b Institut für Anorganische Chemie, 9179 Christian-Albrechts-Universität zu Kiel, Max-Eyth-Str. 2, Kiel 24118, Germany; c Kiel Nano, Surface and Interface Science KiNSIS, 9179 Christian-Albrechts-Universität zu Kiel, Christian-Albrechts-Platz 4, Kiel 24118, Germany

## Abstract

Efficient removal of acidic impurities such as hydrogen chloride (HCl) is essential for hydrogen purification, yet most conventional adsorbents are nonregenerable and require frequent replacement, underscoring the need for scalable, regenerable alternatives. This study evaluates the aluminum-based metal–organic framework (MOF) Al-CAU-60 as a regenerable HCl adsorbent. A reproducible, scalable reflux synthesis was developed for phosphonate-based Al-CAU-60·6HCl, enabling 10 L scale production with high yield (151 g, >96%) and preserved crystallinity. Following neutralization, the MOF was shaped into mechanically robust pellets (Al-CAU-60/PVF) using 10 wt % polyvinyl formal (PVF) as a binder. Structural and chemical integrity were confirmed through scanning electron microscopy–energy-dispersive X-ray spectroscopy (SEM-EDX), powder X-ray diffraction (PXRD), attenuated total reflectance–Fourier transform infrared (ATR-FTIR), and thermogravimetric (TGA) analyses. Under dynamic breakthrough tests (10 bar, 25 °C, 100 ppm of HCl), Al-CAU-60/PVF showed an average HCl uptake of 1.38 mmol/g at the 1 ppm breakthrough and 1.73 mmol/g at saturation. The material retained its performance over seven adsorption–regeneration cycles using only water-triggered desorption, demonstrating an exceptional example of fully regenerable, phosphonate-based MOF sorbents. In contrast, zeolite 13X, while initially more active, lost ∼90% capacity after the first cycle, confirming the superior cyclic stability of Al-CAU-60/PVF. Mechanistic analysis revealed that HCl adsorption proceeds via reversible protonation–deprotonation of phosphonate groups during water-based regeneration.

## Graphical abstract

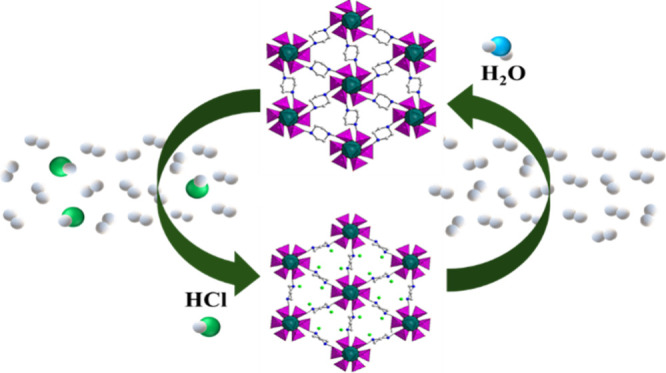

## Introduction

Hydrogen gas is a key player in the transition to clean energy^1,2^ and plays a vital role across various industries, including oil refining,^3^ metal welding,^4,5^ and the production of ammonia,^4^ semiconductors,^6^ and pharmaceuticals.^4,7^ In comparison to conventional fuels, hydrogen offers numerous advantages such as higher energy density, zero emissions during combustion, and flexible storage options (i.e., gas, liquid, or solid).^3,8,9^ Though not readily available in nature, hydrogen can be produced from a variety of feedstocks, including fossil fuels and renewable resources.^10^ In the chemical industry, hydrogen is primarily obtained as a byproduct during the reforming of hydrocarbons, also known as steam methane reforming (SMR).^8,10^ To enhance the process performance, chlorine-promoted catalysts are often used in this process.^11–13^ However, this also leads to the formation of hydrogen chloride (HCl) impurities in the product stream, which pose significant challenges. Apart from contaminating the final product, HCl can cause severe corrosion, fouling, and equipment damage, resulting in safety hazards and environmental risks.^12–15^ Therefore, the effective removal of HCl is critical for both industrial operations and environmental protection.

Several techniques including partial condensation,^16^ chemical reactions,^16,17^ membrane and molecular sieve separation,^16,18^ and high-temperature absorption on mineral based absorbents^19–21^ have been explored for hydrogen purification. However, achieving efficient removal of HCl at high gas velocities and low concentrations is quite challenging. Adsorption at low temperatures offers a selective and energy-efficient alternative and has been proven effective at process conditions.^22,23^ Various adsorbents, such as carbonate-based sorbents,^21^ activated carbon (AC),^24,25^ and zeolites^14,22,26^ have shown potential for HCl adsorption due to their tunable pore structures and high thermal stability. Nevertheless, most of these adsorbents lack regenerability, and once saturated with HCl they typically require replacement, which limits their long-term applicability in industrial processes.^27^

Beyond conventional sorbents, porous materials like metal–organic frameworks (MOFs) are increasingly being considered for the removal of hazardous gases due to their high uptake capacities. For example, an amine functionalized UiO-66 MOF has been reported to adsorb up to 1.4 g/g of nitrogen dioxide and 1.24 g/g of chlorine.^28,29^ Similarly, MFM-300­(Sc) (NOTT-400) demonstrated a high adsorption capacity of 9.4 mmol/g for SO_2_ molecules and facile regeneration via vacuum treatment for 30 min at room temperature.^30^ Another MOF, PCM-75, exhibited a high H_2_S uptake of 8.0 mmol/g, with DMF-triggered H_2_S release and spontaneous crystallization-based regeneration.^31^ For HCl removal, ZIF-8 has shown exceptional performance in trace level removal impurity capture, achieving 1.02 g/g under dynamic process conditions.^23^ However, this unprecedented capacity arises from an irreversible chemical reaction, making the material nonregenerable, and necessitating a complete replacement once its HCl uptake capacity is reached.

Another class of porous materials, metal phosphonates, is gaining research interest due to their high stability and structural diversity, which arise from the phosphonic acid functional group’s ability to adopt multiple coordination modes and protonation states.^32,33^ This versatility enables a wide range of metal-binding configurations, allowing tunable and complex framework structures, making them attractive for adsorption,^34^ catalysis,^35^ and functional device development.^36,37^ In the context of HCl capture, Reichenau et al. discovered a porous aluminum phosphonate MOF, Al-CAU-60·6HCl, which exhibited reversible and rapid desorption of HCl in water.^38^ This MOF with composition [Al_2_H_12_(PMP)_3_]­Cl_6_·6 H_2_O (H_4_PMP = C_6_H_16_N_2_O_6_), which corresponds to 18.3 wt % HCl, is synthesized under highly acidic conditions. It contains trivalent aluminum ions (Al^3+^) and protonated phosphonate groups that maintain the charge balance. When immersed in deionized water, Al-CAU-60·6HCl rapidly deprotonates, converting to its hydrated form Al-CAU-60·9H_2_O and releasing HCl molecules within 2 min, thus highlighting its potential as a regenerable material for HCl removal.

Despite these promising properties, MOFs cannot be directly used in industrial adsorption columns. For practical deployment, two key requirements must be met: reproducible and scalable synthesis routes that deliver material in large quantities,^39^ and shaping strategies that convert powders into mechanically robust forms, such as pellets or extrudates, while preserving their adsorption performance.^40,41^ For HCl removal, however, an additional barrier is the nonregenerative nature of most sorbents, which necessitates frequent replacement of sorbent or chloride guard beds. These are critical for preventing corrosion, maintaining operational efficiency, reducing costs, and ensuring environmental compliance.^42^ To date, very few adsorbents have demonstrated the ability to undergo regeneration, typically requiring thermal treatment (Table S1). Notably, Al-CAU-60·6HCl has shown promising water-triggered regeneration.^37^ However, large-scale synthesis of phosphonate-based MOFs such as Al-CAU-60 remain challenging due to their tendency to form dense nonporous structures, the high anionic charge of phosphonate linkers, and the requirement for strongly acidic conditions to stabilize porous frameworks.^43^ While Reichenau et al. demonstrated Al-CAU-60·6HCl synthesis via conventional and microwave-assisted solvothermal routes at 2 and 20 mL scale,^38^ scaling up to large volume production will require both technical innovations and engineering solutions.

To enable the practical, large-scale application of Al-CAU-60·6HCl for HCl removal, this study develops and evaluates scalable synthesis strategies coupled with shaping approaches to produce mechanically robust pellets while preserving adsorption performance and water-triggered regeneration capability. Three synthesis methods i.e., conventional and microwave-assisted solvothermal, and synthesis under reflux conditions are examined. For structuring, the extrusion-crushing-sieving (ECS) approach is used, in which fine powders or crystals are agglomerated into larger granules or pellets using a binder, such as clay or polymer, and later crushed and sieved to the desired size. A precedent for this strategy was demonstrated by Cousin-Saint-Remi et al., where authors systematically analyzed 55 binder formulations for shaping ZIF-8 MOF into pellets and identified polyvinyl formal (PVF) as the most effective binder.^44^ PVF-based ZIF-8 pellets exhibited high mechanical strength and acid/base resistance while retaining equilibrium adsorption properties, with no adverse effects from the binder.

Building on these findings, this work aims to assess the feasibility of employing Al-CAU-60 as an easily regenerable adsorbent for chloride guard beds. Al-CAU-60 crystals are synthesized via scalable methods and shaped into pellets using PVF as a binder, with different formulations tested to optimize mechanical strength and adsorption efficiency. The cyclic HCl removal performance of these structured Al-CAU-60 pellets is assessed via breakthrough experiments under dynamic process conditions, followed by water-triggered HCl desorption for regeneration. To understand the underlying HCl adsorption mechanism, the structural and chemical properties of the Al-CAU-60 pellets before and after HCl adsorption/desorption are extensively characterized using scanning electron microscopy (SEM) coupled with energy-dispersive X-ray spectroscopy (EDS), X-ray diffraction (PXRD), Fourier transform infra-red spectroscopy (FT-IR), and thermogravimetric analysis (TGA). Finally, a comparative study with commercial zeolite 13X is performed to assess the practical applicability of Al-CAU-60 in industrial gas applications.

## Experimental Section

### Materials and Methods

AlCl_3_·6H_2_O (99%, Sigma-Aldrich), anhydrous piperazine (98%, TCI chemicals), polyvinyl formal (VWR), and dimethylformamide (99%, Grüssing) were used without further purification. The *N*,*N*′-piperazinebis­(methylenephosphonic acid) (H_4_PMP) linker was synthesized by the well-established single-step reaction using anhydrous piperazine (98%, TCI chemicals), an aqueous formaldehyde solution (37 wt %, in water, Sigma-Aldrich), and phosphorous acid (99%, Sigma-Aldrich) as the starting materials and hydrochloric acid (37 wt %, VWR) as the catalyst. A detailed description of the synthesis of *N*,*N*′-piperazinebis­(methylenephosphonic acid) (H_4_PMP) is provided in the Supporting Information (Section S2.2).

### Al-CAU-60·6HCl Synthesis Routes

Three synthesis approaches were employed for the preparation of Al-CAU-60·6HCl. The conventional and microwave-assisted solvothermal routes were adapted from literature procedures to ensure comparability with previous reports, while synthesis under reflux conditions was developed in this study to address scalability limitations. The latter enables batch synthesis in reactors ranging from 250 mL to 10 L, providing a more practical pathway for large-scale production.

### Conventional Solvothermal Synthesis

Al-CAU-60·6HCl was synthesized with slight modifications from the procedure reported in the literature.^38^ In a typical synthesis, AlCl_3_·6H_2_O (1851 mg, 0.75 mmol), H_4_PMP (361 mg, 1.32 mmol), H_2_O (6.42 mL), and concentrated HCl (2.08 mL, 37 wt %) were combined in a 30 mL Teflon-lined reactor, sealed in a steel autoclave, and heated to 160 °C at 0.75 °C/min for 24 h. The mixture was then cooled to 25 °C over 3 h. After the reaction, the solid product was collected by filtration, thoroughly washed with aqueous HCl (*c* = 2 mol/L), and dried at room temperature.

### Microwave-Assisted Solvothermal Synthesis

Microwave-assisted synthesis of Al-CAU-60·6HCl was carried out following Reichenau et al.^38^ A mixture of AlCl_3_·6H_2_O (181 mg, 0.75 mmol), H_4_PMP (361 mg, 1.32 mmol), deionized water (8.1 mL), and concentrated HCl (1.9 mL, 37 wt %) was heated in a microwave glass vials at 160 °C for 3 h. The resulting solid was recovered by filtration, thoroughly washed with aqueous HCl solution (*c* = 2 mol/L), and dried at room temperature.

### Large-Scale Synthesis of Al-CAU-60·6HCl under Reflux Conditions

A large-scale synthesis route under reflux conditions was developed to enable production of Al-CAU-60·6HCl under highly acidic conditions. Batch sizes from 250 mL up to 10 L were systematically evaluated (Table S2). For a representative 10 L synthesis (Figure 1a), AlCl_3_·6H_2_O (62.4 g, 0.25 mol) was dissolved in a HCl/H_2_O mixture consisting of HCl (37 wt %, 787.5 mL) and deionized water (3150 mL). After complete dissolution of the metal salt, H_4_PMP (120 g, 0.43 mol) was added to the reaction mixture. The mixture was then refluxed under continuous stirring at 130 °C for 14 h in the fume hood. Upon completion, the resulting white solid was recovered by filtration, washed with 200 mL of aqueous HCl (*c* = 2 mol/L), and subsequently air-dried at room temperature (Figure 1b). The reaction parameters i.e., temperature and reaction time were optimized to maximize yield, with variations primarily attributed to washing and filtration losses. The reaction yield was calculated on a metal basis.

**Figure 1:**
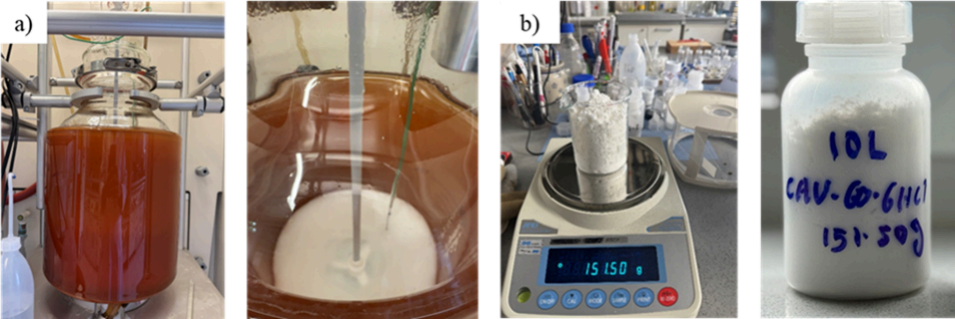
Photograph of (a) 10 L batch reactor for Al-CAU-60·6HCl synthesis under reflux conditions and (b) dried Al-CAU-60·6HCl powder stored in a polypropylene (PP) bottle.

### Al-CAU-60·9H_2_O Pellet Formulation

Formulation of MOF crystals into mechanically stable particles is crucial for their use in adsorptive separation processes. Prior to pelletization, Al-CAU-60·6HCl was converted into its hydrated form Al-CAU-60·9H_2_O by controlled NaOH treatment, followed by washing and drying (details provided in the Supporting Information (Section S2.3)). For clarity, in this work the term Al-CAU-60 refers to Al-CAU-60·9H_2_O, unless stated otherwise.

Al-CAU-60 pellets were synthesized following the approach outlined by Cousin-Saint-Remi et.al.,^44^ using a polyvinyl formal (PVF) binder. In a typical procedure, 1.0 g of Al-CAU-60 crystals was mixed with a PVF binder dissolved in dimethylformamide (DMF) (5 mL, 80 °C, 40 min) to obtain a homogeneous slurry. The resulting slurry was then extruded through a 2 mm syringe nozzle onto a glass plate, dried overnight, and subsequently crushed and sieved to obtain particles within a specific size range (Figure 2). The binder-to-solid mass fraction was systematically varied between 1.0 and 10.0 wt % to optimize mechanical stability and adsorption performance. The binder content in the final pellets used for breakthrough experiments was also verified by energy dispersive X-ray spectroscopy (EDX). Consult Section S3.3 from the Supporting Information for detailed information.

**Figure 2:**
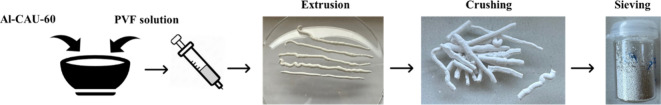
Formulation route to obtain Al-CAU-60/PVF pellets.

### Stability and Recovery of Al-CAU-60 Pellets after Water-Triggered Regeneration

In addition to evaluating HCl removal performance through adsorption tests, it is essential to assess the recovery and stability of Al-CAU-60 pellets after regeneration with an aqueous solution. Ensuring minimal material loss during regeneration is essential for maintaining long-term efficiency in industrial applications. To evaluate the stability of the structured pellets, two tests, namely single-pellet crush strength and a controlled water immersion test were conducted. Crush strength measurements were performed using a SAUTER TVL test stand, where individual pellets (1.0–1.1 mm) and extrudates (2.0–2.2 mm) were subjected to an increasing axial load until fracture. For the water immersion test, sieved pellets (200–280 μm), obtained from multiple batches, were immersed in deionized water under continuous stirring for 2 h. Following this, the pellets were filtered, dried, and resieved to determine any changes in their structural integrity. This test was repeated five times to simulate repeated regeneration cycles. For thermal stability, thermogravimetric analysis was performed (consult Section 2.6 for details).

### Breakthrough Experimentation

Gas phase breakthrough experiments were carried out with packed columns placed in an in-house made setup composed of coated components to minimize hydrogen chloride (HCl) adsorption on the setup material surface (Figure 3a). Columns (i.e., coated stainless steel tubes of 5.0 cm length and 0.216 cm internal diameter) were packed with ∼100 mg of pellets (200–280 μm) surrounded by quartz wool (Figure 3b) under ambient conditions. Experiments were performed at room temperature (25 °C) and at a total pressure of 10 bar.

**Figure 3:**
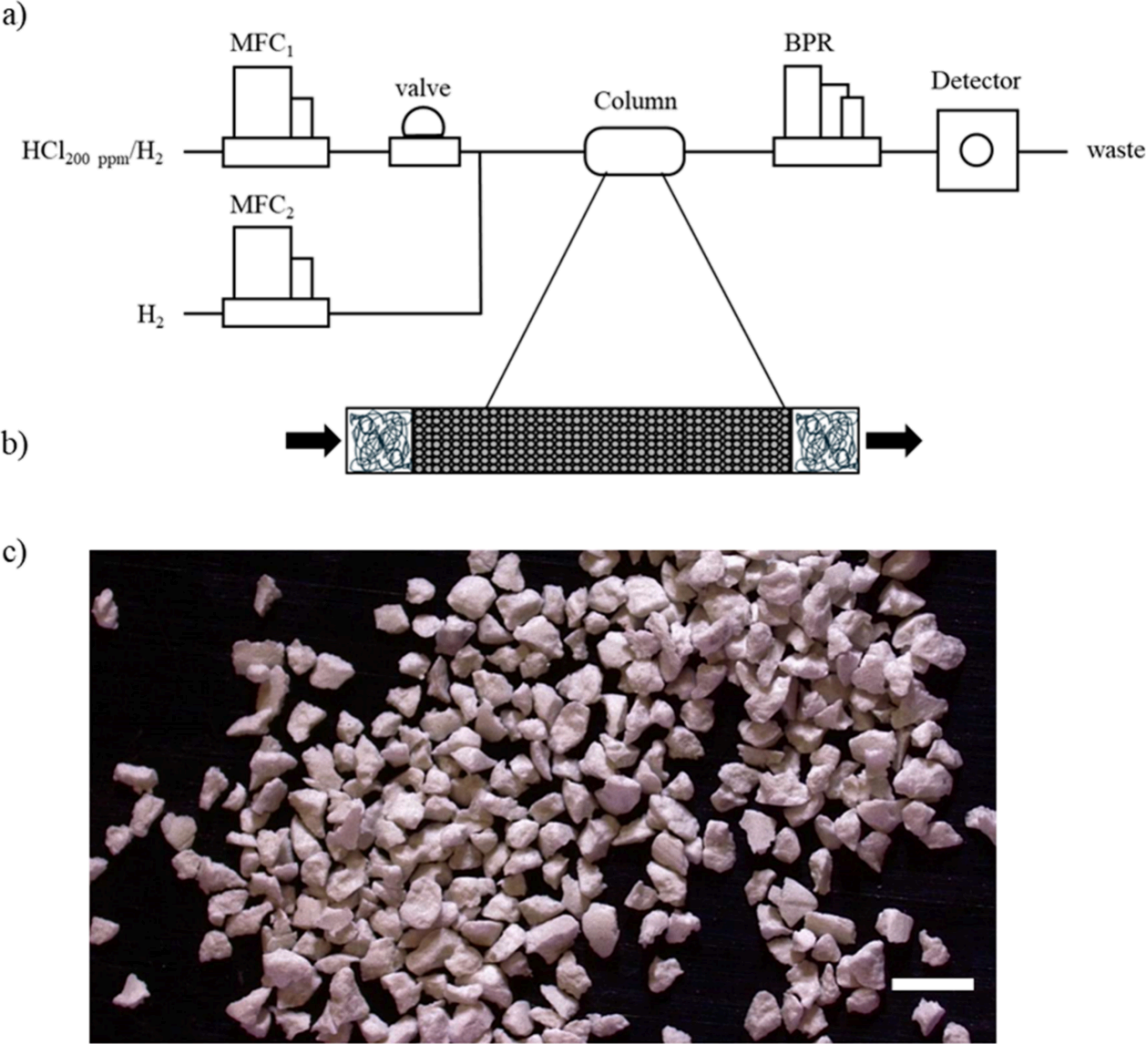
(a) Scheme of the HCl breakthrough setup with mass flow controllers (MFC), backpressure regulator (BPR), and online laser gas detector, (b) packed column filled with quartz wool and Al-CAU-60 pellets (dark), and (c) optical microscopy image of Al-CAU-60 pellets (scale bar: 500 μm).

Before connecting the packed columns to the setup, the setup was first saturated with the HCl/H_2_ mixture under the selected experimental conditions, followed by a quick flush with pure hydrogen gas to remove HCl that was not bounded on the setup surface. Afterward, the packed column was connected to the setup and contacted with HCl at room temperature by changing the feed from pure hydrogen gas to a hydrogen gas stream containing 100 ppm of HCl. The gas composition was controlled by calibrated mass flow controllers, mixing pure hydrogen gas and hydrogen gas containing HCl (see above). The gas velocity was set at 0.30 m/s. HCl was detected at the column outlet with an online calibrated laser detector (Axetris, Switzerland). Breakthrough curves were obtained by plotting the HCl concentration, detected at the column outlet, as a function of time. The amount of HCl adsorbed, *q* (g/g) was calculated from the mass balance over the column, using eq 1:

$$q=\frac{\left(F\tau -\varepsilon V\right)}{m_{\mathrm{ads}}}\times \frac{yp}{RT}M$$ 1

with *F*, τ, ε, *V*, *m*_ads_, *y*, *p*, *R*, *T*, and *M* representing the (volumetric) feed flow rate (mL/min), the average breakthrough time (min), the bed porosity, the column volume (mL), the adsorbent mass (g), the HCl volume fraction in the feed, the total pressure (bar), the gas constant (J/K/mol), the temperature (K) and the molar mass of HCl (g/mol). The feed volumetric flow rate (and gas velocity) was calculated from the column dimensions, the pressure in the column and the volumetric flow rate at the setup outlet. The latter was measured with a bubble flow meter. The average breakthrough time was calculated from eq 2:

$$\tau =\int_{0}^{\infty }\left(1-\frac{c}{c_{0}}\right)dt$$ 2

with *c* and *c*_0_ denoting the HCl concentration at the column outlet and the feed concentration, respectively. In brief, the average breakthrough time is defined as the time integral of the normalized adsorption capacity of the bed, calculated from the outlet concentration profile according to eq 2. Physically, this quantity represents the mean residence time of HCl molecules in the adsorbent bed under dynamic conditions. The upper integration limit corresponds to bed saturation, defined experimentally as the point where the outlet concentration equals the inlet concentration (*c*/*c*_0_ → 1). This definition is commonly used in breakthrough analysis to quantify the overall adsorption performance of a fixed bed from the complete breakthrough curve. The reproducibility of the experimental approach is presented in the Supporting Information (Section S4). Additionally, the adsorption capacity at 1 ppm breakthrough is determined by integration of eq 2 until 1 ppm is detected at the column outlet.

To evaluate the reusability of Al-CAU-60 pellets, cyclic HCl adsorption–desorption breakthrough experiments were performed using two regeneration approaches: thermal and water-triggered. In the thermal regeneration method, the HCl-saturated pellets were heated to 250 °C under a helium flow while packed in the adsorption column. For the water-triggered route, the pellets were immersed in 25 mL deionized water after HCl adsorption. The pH of the solution was monitored during immersion, and once it stabilized, sodium hydroxide (NaOH, *c* =0.1 mol/L) was added to neutralize the released acid. Following regeneration, the pellets were recovered by filtration and subjected to a two-step drying process: an initial drying at 60 °C for 5 min, followed by ambient air drying for 2 h. The regenerated pellets were then packed and retested in HCl breakthrough experiments under the same conditions as the initial adsorption test (*vide supra*) to assess the material’s performance over multiple cycles. In a continuous industrial process, this ex-situ regeneration could be replaced with in situ regeneration by flushing the column with water followed by compressed air drying to improve efficiency.

### Characterization Techniques

The bulk textural and chemical (composition) properties were investigated via scanning electron microscopy (SEM) coupled with energy dispersive X-rays spectroscopy (EDX) using a JSM-IT300 JEOL device under vacuum (ca. 10^–6^ Torr) at a voltage of 20 kV. Al-CAU-60 particles were fixed with carbon tape on a sample holder and coated with a silver layer (∼15 nm). The atomic percentages detected by EDX analysis in the Al-CAU-60 particles were used to calculate the chemical composition. Following the repeated measurements, the average deviation for the EDX analysis is estimated at 5% and maximum 11% in relative standard deviation within a same sample.

The crystallinity of the different samples was explored via powder X-ray diffraction (PXRD) under ambient conditions. A Bruker device (D8 Advance Eco diffractometer) was used with Cu Kα radiation (40 kV, 25 mA) and a LynxEye XE-T (PSD opening 3.3°) detector in the range of 5–70° 2θ (with 0.015° step size and time 0.5 s/step). The attenuated total reflectance–Fourier transform infrared (ATR-FTIR) spectra were recorded using a Bruker Vertex 70 V device over a frequency range of 500–4000 cm^–1^. Samples were mounted under ambient conditions and analyzed under reduced pressure (i.e., less than 1 hPa). The material behavior upon heating was analyzed by thermogravimetric analysis (TGA) with a SENSYS Evo instrument (SETARAM Instrumentation). About 10 mg of material was placed in the sample holder and subjected to a gradual increase in temperature (0.1 K/min) from 25 to 450 °C under a pure helium stream (100 N mL/min). A measurement was also conducted at a constant temperature of 25 °C. The sample weight was recorded as a function of time.

## Results and Discussion

This study aims to assess the potential of the aluminum phosphonate MOF, Al-CAU-60·9H_2_O, for cyclic HCl adsorption under practically relevant conditions, focusing on its scalable synthesis and PVF-based pelletization into mechanically robust forms. The pellets were evaluated under dynamic breakthrough conditions for adsorption performance and cyclic stability, characterized to elucidate the HCl adsorption mechanism in pellet form, and benchmarked against commercial 13X zeolite.

### Al-CAU-60·6HCl Synthesis Strategies for Scalable Production

The large-scale production of Al-CAU-60·6HCl poses challenges due to the limitations of conventional synthesis routes. While conventional and microwave-assisted solvothermal methods reported by Reichenau et al.,^38^ are effective for small-scale synthesis, with microwave synthesis notably reducing reaction time, both approaches are challenging to scale up for the quantities required in industrial applications.^45,46^ To overcome this limitation, a synthesis strategy under reflux conditions was developed in this work, offering a practical route to produce high amounts of Al-CAU-60·6HCl while maintaining its structural integrity.

The feasibility of large-scale MOF synthesis is primarily determined by the accessibility and scalability of the precursor materials, particularly the choice of metal salts and ligands.^47^ For the synthesis of Al-CAU-60·6HCl, commercially available AlCl_3_·6H_2_O and the ligand *N*,*N*′-piperazinebis­(methylenephosphonic acid) (H_4_PMP) were used, with H_4_PMP prepared in-house from piperazine in high yield (110 g, ∼85%, Section S2.2). Preliminary trials under conventional solvothermal, microwave-assisted solvothermal, and synthesis under reflux conditions confirmed that crystalline Al-CAU-60·6HCl ([Al_2_H_12_(PMP)_3_Cl_6_]·6H_2_O) could be obtained with consistent crystallinity in all cases (Figure S1b), demonstrating the versatility of the reaction conditions. Subsequently, the method under reflux conditions was systematically scaled from 250 mL to 10 L to evaluate its suitability for large-scale production. For each batch, the crystallinity of the resulting Al-CAU-60·6HCl remained consistent, as confirmed by PXRD (Figure S1c,d). The reaction yield, calculated on a metal basis, exceeded 96% in all cases, with minor variations primarily due to losses during washing and filtration (Table S2). The mass of MOF obtained increased proportionally with reaction volume, yielding 4.45 g at 250 mL, 8.80 g at 500 mL, 17.40 g at 1 L, and 151.5 g at 10 L. For the 10 L batch, minor adjustments were made—raising the oil bath temperature to 130 °C and extending the reflux time to 14 h—to ensure reproducibility and high yield (97.6%). This successful demonstration of consistent crystallinity, high yield, and proportional scaling highlights the robustness of the route under reflux conditions. It provides a practical foundation for producing Al-CAU-60·6HCl for subsequent pelletization and HCl adsorption studies.

### Al-CAU-60/PVF Pellets: Synthesis, Characterization, and Stability Evaluation

Following the successful large-scale synthesis, the next step focused on shaping Al-CAU-60·6HCl into mechanically robust particles of sufficiently large size, a critical requirement for its practical implementation in adsorption-based HCl removal. Before shaping, Al-CAU-60·6HCl was neutralized to Al-CAU-60·9H_2_O using a controlled NaOH treatment, followed by thorough washing and drying (∼95% yield). The converted MOF was analyzed by PXRD, which confirmed the phase transformation (Figure S2a). Further details on this conversion are provided in Section S2.3 of the Supporting Information, and for clarity, the term Al-CAU-60 is used throughout this work to denote Al-CAU-60·9H_2_O unless stated otherwise. Based on the approach of Cousin-Saint-Remi et.al.,^44^ Al-CAU-60 was structured into PVF-based pellets via an extrusion–crushing–sieving (ECS) method and three binder loadings i.e., 1.0, 5.0, and 10.0 wt % were evaluated. Pellets prepared with 1.0 wt % PVF exhibited poor cohesion between the Al-CAU-60 crystals and the binder, resulting in extrudates that collapsed after the drying step. Increasing the binder content to 5.0 wt % improved the agglomeration; however, the pellets remained mechanically weak and fell apart during handling. The optical microscope and SEM images of these pellets are exhibited in Figure S3, clearly showing the insufficient structural integrity and fragmentation of the pellets.

To address this, the binder content was further increased to 10.0 wt %, resulting in robust pellets (Figure 3c). The single-pellet crush strength of Al-CAU-60/PVF was measured to be 4.5 N, approximately 2-fold lower than that of PVF based 13X pellets and comparable with ZIF-8/PVF pellets^48^ (Table S3 and Section S3.3). Despite the lower strength relative to the commercial benchmark, the MOF pellets exhibit sufficient mechanical stability for fixed-bed operation while requiring only a limited amount of binder.

Next, three batches of Al-CAU-60/PVF pellets with 10.0 wt % binder were synthesized to ensure reproducibility. The resulting materials were then characterized using various methods, as explained below (Figure 4 and Figure S4 and Table S4). First, the optical microscope image of Al-CAU-60 pellets with 10.0 wt % PVF (Figure 3c) and the SEM images of the pristine powder (crystals) and the shaped pellets (Figure S4a,b) indicated that the primary crystal morphology and overall pellet structure were well preserved after shaping. Energy dispersive X-ray (EDX) analysis confirmed the elemental composition of the pellets. Characteristics peaks corresponding to nitrogen (0.392 keV), oxygen (0.526 keV), aluminum (1.48 keV), and phosphorus (2.01 keV) were observed, consistent with the elemental profile of the pristine Al-CAU-60 crystals (Figure 4a and Figure S5). A peak at 2.98 keV was also present, corresponding to the Ag coating applied to the samples prior to EDX analysis. The average binder content was calculated as 11.7 wt % (Section S3.4). Small deviations in binder content between batches may occur due to the difficulty in controlling the amount of binder which agglomerates with the Al-CAU-60 crystals and the amount remaining after the crushing step.

**Figure 4:**
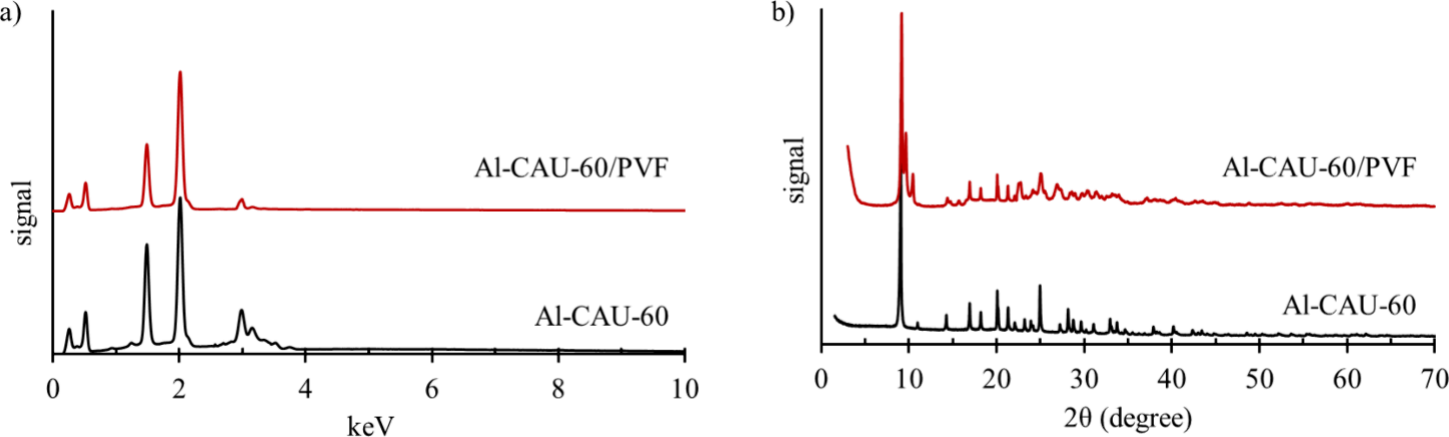
Al-CAU-60 crystals (black, bottom) and Al-CAU-60/PVF pellets (red, top): (a) energy-dispersive X-ray (EDX) spectra and (b) X-ray diffraction (PXRD) pattern.

The PXRD patterns of the pellets retained the characteristic reflections of Al-CAU-60 at 9.05°, 16.9°, 18.2°, and 20.1°,^38^ confirming that the crystalline framework remained intact after pelletization (Figure 4b). Two additional reflections at 9.7° and 10.5° were seen, likely arising from structural changes during storage (Figure S2b). This behavior is consistent with flexible MOFs, which can undergo structural modifications in response to external stimuli such as temperature, mechanical forces, or guest loadings.^49,50^ The overall preservation of crystallinity was further supported by ATR-FTIR spectroscopy (Figure S6). Both pellets and pristine crystals displayed identical vibrational features, including P–O stretching vibrations at 920 cm^–1^ and 970 cm^–1^, C–O stretching at 1080 cm^–1^ and N–H stretching bands in the 3010–3020 cm^–1^ range (Figure S6).^38,51^ These results confirm that the PVF-based shaping approach preserves both the chemical structure and crystallinity of Al-CAU-60, making the resulting pellets suitable for adsorption-based applications.

The thermal stability of the Al-CAU-60/PVF pellets was assessed using thermogravimetric analysis (TGA), with results for both pristine Al-CAU-60 crystals and Al-CAU-60/PVF pellets shown in Figure S7. The TGA profiles of both materials follow similar trends. An initial mass loss, 11.5% for the Al-CAU-60 crystals and 13% for Al-CAU-60/PVF pellets, was observed between 25 and 175 °C, attributed to the removal of solvent molecules like H_2_O, DMF, from the composite. Subsequently, both materials underwent exothermic decomposition of the composite, starting around 300 °C and completing near 500 °C. These findings are consistent with the previously reported thermal behavior for Al-CAU-60.^38^ In addition to thermal stability, the mechanical integrity of the shaped pellets under aqueous conditions was evaluated to assess their durability during water-triggered regeneration. Structured Al-CAU-60/PVF pellets (ca. 1.0 g) were subjected to five consecutive water immersion cycles, as described in Section 2.4. The cumulative mass loss after these cycles was about 3 wt % (Table S4). The preserved structural integrity of the pellets was further confirmed by optical microscopy and SEM images shown in Figure S4c,d. The combined results from TGA and immersion test indicate that Al-CAU-60/PVF pellets exhibit good thermal and mechanical stability under regeneration conditions, with minimal degradation. This highlights their strong potential for long-term use in chloride guard beds, where repeated regeneration cycles are necessary to ensure process efficiency and minimize operational downtime.^42^

### HCl Removal Performance

The hydrogen chloride (HCl) removal performance of the Al-CAU-60/PVF pellets was evaluated under dynamic process conditions using a packed-bed configuration and a hydrogen stream containing 100 ppm of HCl, corresponding to a partial pressure of 1.03 mbar, at 10 bar total pressure. The bulk density of the pellets was calculated as 0.41 g/mL. Figure 5a shows the HCl breakthrough profile obtained with column packed with Al-CAU-60/PVF pellets (200–280 μm). The breakthrough profile exhibits a sharp exit and broader HCl elution profile, with breakthrough occurring around 38 min and saturation reached after 120 min. This indicates that Al-CAU-60/PVF pellets effectively and selectively adsorb HCl from the hydrogen stream. The broadening of the elution profile suggests mass (and/or heat) transfer resistance or a limitation in HCl binding kinetics.^22^ From the mass balance calculations, Al-CAU-60/PVF pellets exhibited a breakthrough capacity of 1.29 mmol HCl/g_Al‑CAU‑60/PVF_ (defined at 1 ppm outlet concentration), which does not deviate substantially from that obtained at saturation, i.e., 1.60 mmol HCl/g_Al‑CAU‑60/PVF_. This is particularly relevant, as the purification efficiency of fixed-bed adsorbers is typically defined by the capacity at initial breakthrough.^40^

**Figure 5:**
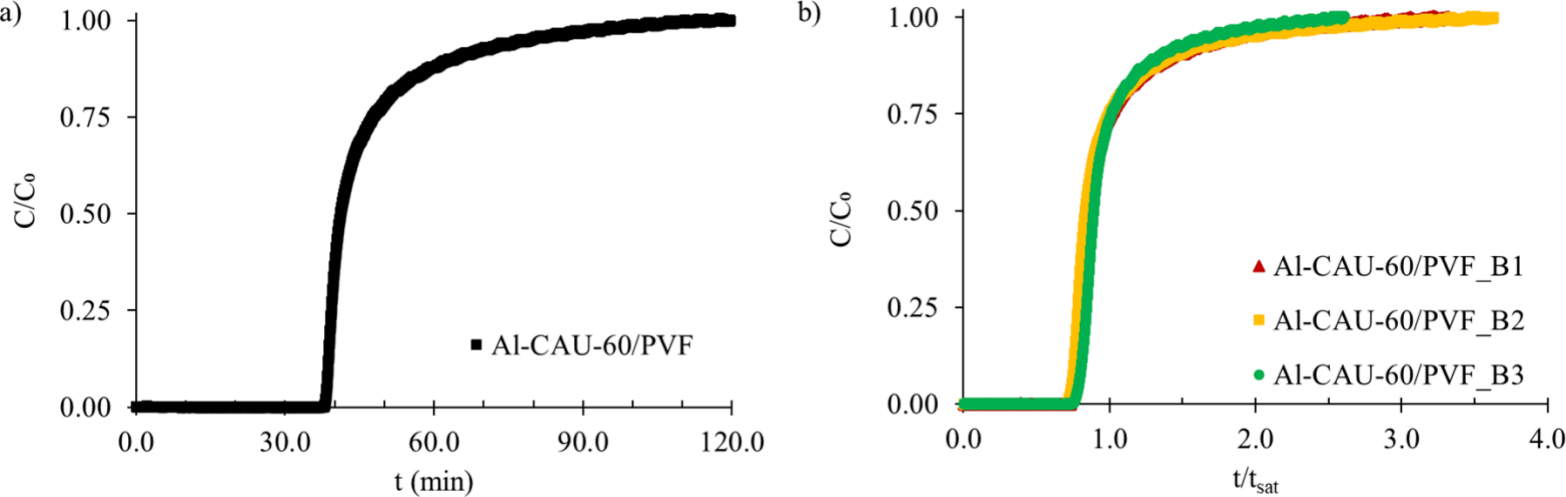
(a) HCl breakthrough curves measured with a packed column containing Al-CAU-60/PVF pellets (200–280 μm) at 25 °C, 0.30 m/s gas velocity, 1.03 mbar HCl partial pressure (100 ppm), and 10 bar total pressure and (b) HCl breakthrough profiles for beds packed with three batches of Al-CAU-60/PVF pellets (200–280 μm) as a function of dimensionless time defined as the ratio of operating time (*t*) to stoichiometric (average) breakthrough time (*t*_sat_) obtained at 25 °C, 0.30 m/s gas velocity, 1.03 mbar HCl partial pressure, and 10 bar total pressure.

To ensure reproducibility and consistency, breakthrough profiles from three independently synthesized pellet batches were compared (Figure 5b and Figure S8), showing negligible variation in performance (Table S5). It is worth noting that the shaped pellets exhibited an approximate 11% reduction in adsorption capacity compared to pristine, binderless Al-CAU-60 powder (Figure S9 and Table S4), which corresponds to the amount of PVF binder and possible partial pore blocking during shaping. Nonetheless, the Al-CAU-60/PVF pellets maintain high HCl removal efficiency while offering the mechanical strength necessary for industrial operation in fixed-bed systems.

### Cyclic Performance

Regeneration is a critical factor that determines the reusability and long-term efficiency of adsorbent materials. To evaluate the regenerability of Al-CAU-60/PVF pellets following HCl adsorption, two different approaches were explored: a thermal regeneration route and a water-triggered regeneration route (see Section 2.5 for experimental details). After the first HCl adsorption cycle, the pellets were regenerated using each method and then subjected to a second breakthrough experiment under identical process conditions to the initial test. Figure S10a shows the breakthrough curves before and after thermal regeneration at 250 °C. A significant drop in HCl adsorption capacity was observed, with the saturation capacity decreasing from 1.60 mmol/g to just 0.12 mmol/g. This sharp decline indicates that thermal regeneration at 250 °C was ineffective, likely due to a substantial portion of the active adsorption sites remaining partially saturated with HCl molecules and/or due to partial structural degradation of the framework.^38^ To evaluate this further, TGA was conducted on HCl-loaded samples, while PXRD was performed on pellets subjected to thermal treatment at 250 °C after adsorption. TGA analysis (Figure S10b) revealed an additional mass loss between 225 and 350 °C, which may correspond to minor HCl release and/or partial structural degradation. Consistent with this, the PXRD pattern of thermally treated pellets (Figure S11) showed a reflection shift to 8.5°, indicating framework alteration due to residual HCl^38^ and confirming incomplete regeneration of the MOF. A more detailed discussion of the thermal behavior of Al-CAU-60/PVF pellets before and after HCl exposure is provided in Section 3.5. Taken together, these results confirm that thermal treatment alone is insufficient for complete regeneration of Al-CAU-60/PVF pellets, thus highlighting the need for an alternative regeneration strategy.

Next, a water-triggered desorption approach was applied to regenerate the HCl-loaded Al-CAU-60/PVF pellets. After the first adsorption cycle, the pellets were dispersed in deionized water to initiate desorption, and the pH of the solution was monitored throughout the immersion process (see Section 2.5 for experimental details). The regenerated pellets were then subjected to a second HCl breakthrough experiment under the same conditions as the initial test (*vide supra*). Figure 6 presents the HCl breakthrough profiles before (Cycle 1) and after regeneration (Cycle 2), along with the corresponding pH measurements recorded during the desorption process. Interestingly, a slight improvement of about 8% in HCl removal performance (adsorption capacity) was observed after the regeneration, with the saturation capacity increasing to 1.72 mmol/g and the breakthrough capacity reaching 1.40 mmol/g for Cycle 2 (Figure 6a). During immersion of the HCl-saturated adsorbent particles in water, a rapid drop in pH was observed, from 6.9 to 2.6 within the first minute, followed by stabilization at pH 2.3 (Figure 6b). This change corresponds to the release of approximately 0.125 mmol of HCl, which matches the amount adsorbed during the initial cycle, thus confirming full regeneration achieved via water-triggered regeneration method. Following this successful regeneration, a total of seven adsorption–desorption cycles was conducted.

**Figure 6:**
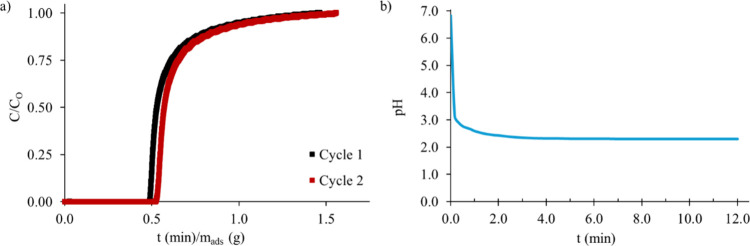
(a) HCl breakthrough profiles as a function of mass-normalized time (*t*/*m*_ads_, where *t* is the operating time and *m*_ads_ is the mass of adsorbent loaded) for beds packed with Al-CAU-60/PVF pellets before (Cycle 1) and after water-triggered desorption at 25 °C (Cycle 2); (b) pH of the solution measured during dispersion of Al-CAU-60/PVF pellets after Cycle 1. Breakthrough experiments were conducted at 25 °C, 0.30 m/s gas velocity, 1.03 mbar HCl partial pressure, and 10 bar total pressure.

Figure 7 displays the HCl adsorption capacities and corresponding breakthrough curves plotted as a function of dimensionless time and Figure S12 shows the pH evolution during dispersion of HCl-saturated pellets in deionized water over multiple cycles. As seen from the first regeneration cycle, there was no decline in adsorption performance. The pellets consistently exhibited an average saturation capacity of 1.73 mmol/g and an average breakthrough capacity of 1.38 mmol/g across all cycles. As described earlier in Section 3.2, the adsorbent showed a minor mass loss (∼4%) after seven cycles. These results demonstrate the excellent reusability and stability of Al-CAU-60/PVF pellets for reversible HCl removal under practically relevant conditions.

**Figure 7:**
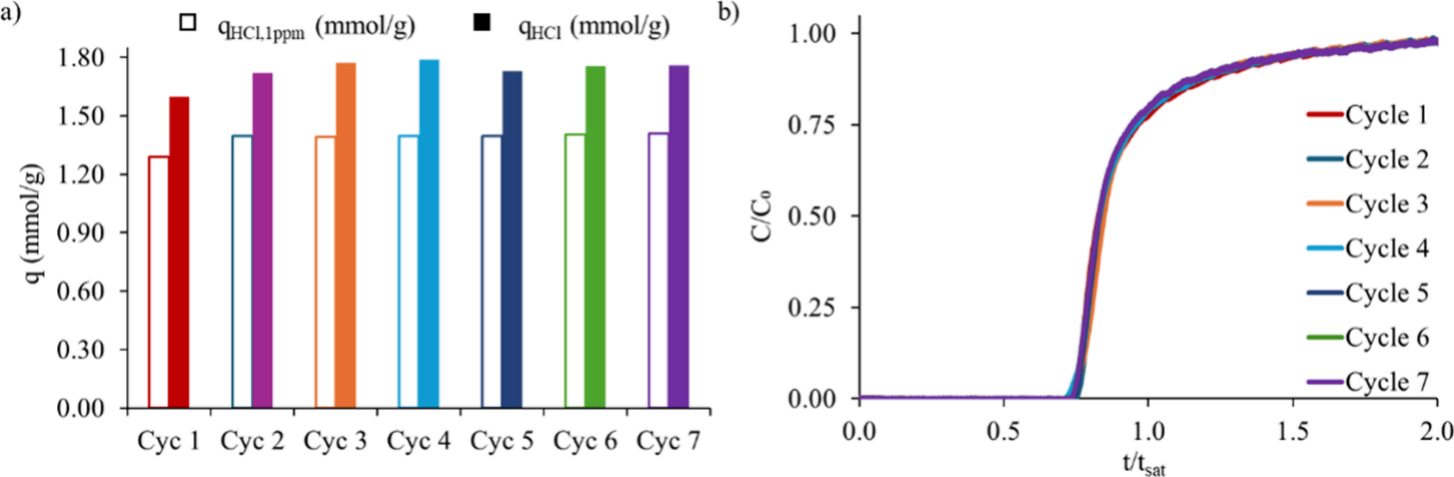
(a) HCl adsorption capacities at saturation (filled bars) and at 1 ppm breakthrough point (empty bars), obtained from breakthrough curves; (b) breakthrough profiles plotted as a function of dimensionless time, defined as the ratio of operating time (*t*) to stoichiometric (average) breakthrough time (*t*_sat_) for Al-CAU-60/PVF pellets over multiple cycles. Breakthrough curves were recorded at 25 °C, 0.30 m/s gas velocity, 1.03 mbar HCl partial pressure, and 10 bar total pressure.

### HCl Adsorption and Water-Triggered Deprotonation Mechanism

To gain further insight into the HCl adsorption mechanism and the subsequent deprotonation during aqueous regeneration, Al-CAU-60/PVF pellets were characterized in three distinct states: before HCl exposure (as-synthesized pellets, S1), after HCl adsorption (S2), and following water-triggered regeneration (S3).

Figure 8 summarizes the morphological, elemental, structural, thermal, and chemical changes observed across the three sample states. Starting with morphology, SEM images revealed morphological changes upon HCl adsorption. Compared to the as-synthesized pellets (S1), which were composed of fine crystals (Figure S4b), the HCl-loaded sample (S2) exhibited increased surface roughness (Figure 8a), indicating possible structural modification due to HCl interaction.^22,23^ The EDX analysis confirmed the presence of chlorine in the S2 sample, with a characteristic Cl peak at 2.62 keV (Figure 8b) and complementary elemental mapping (Figure S13). The average chlorine content was determined to be 6.18 wt %, closely matching the 6.30 wt % adsorption capacity obtained from breakthrough experiments (consult Section 3.3), thereby validating the material’s high affinity for HCl. PXRD analysis further supported these findings (Figure 8c). HCl exposure induced clear changes in the diffraction pattern, including new reflections and a peak shift from 9.10° to 8.50°, suggesting framework alteration. After water-triggered regeneration, the peak reverted back to 9.10° (Figure 8c), demonstrating the reversible structural flexibility of the framework in its pelletized form (Figure 9). This reversible behavior is consistent with the findings of Reichenau et al., where authors reported the coexistence of two phases, i.e., a six HCl form and Al-CAU-60·4HCl, at low pH (pH < 3).^38^ Complementary ATR-FTIR spectra corroborated this behavior. Peaks around 950 cm^–1^ and 1200 cm^–1^, characteristic of P–OH groups,^38,51^ were observed in sample S2 but absent in both S1 and S3 (Figure S14), confirming reversible protonation and deprotonation during the adsorption–regeneration cycle (Figure 9).

**Figure 8:**
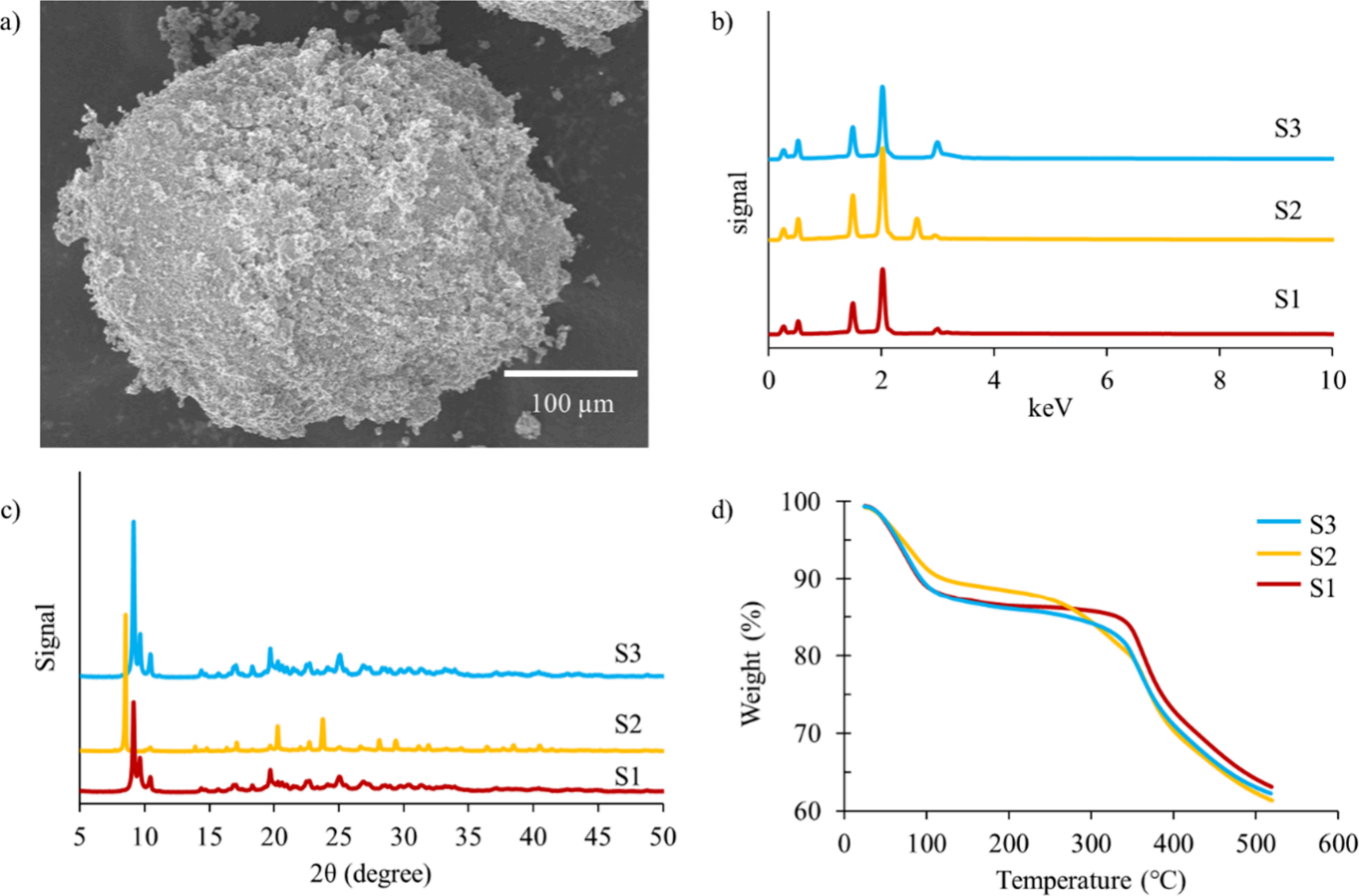
(a) SEM image of Al-CAU-60/PVF pellets after HCl adsorption (S2); (b) EDX-spectra, (c) PXRD-patterns, and (d) TGA-curves of Al-CAU-60/PVF pellets before HCl exposure (S1, bottom), after HCl adsorption (S2, middle), and after water-triggered regeneration (S3, top).

**Figure 9:**
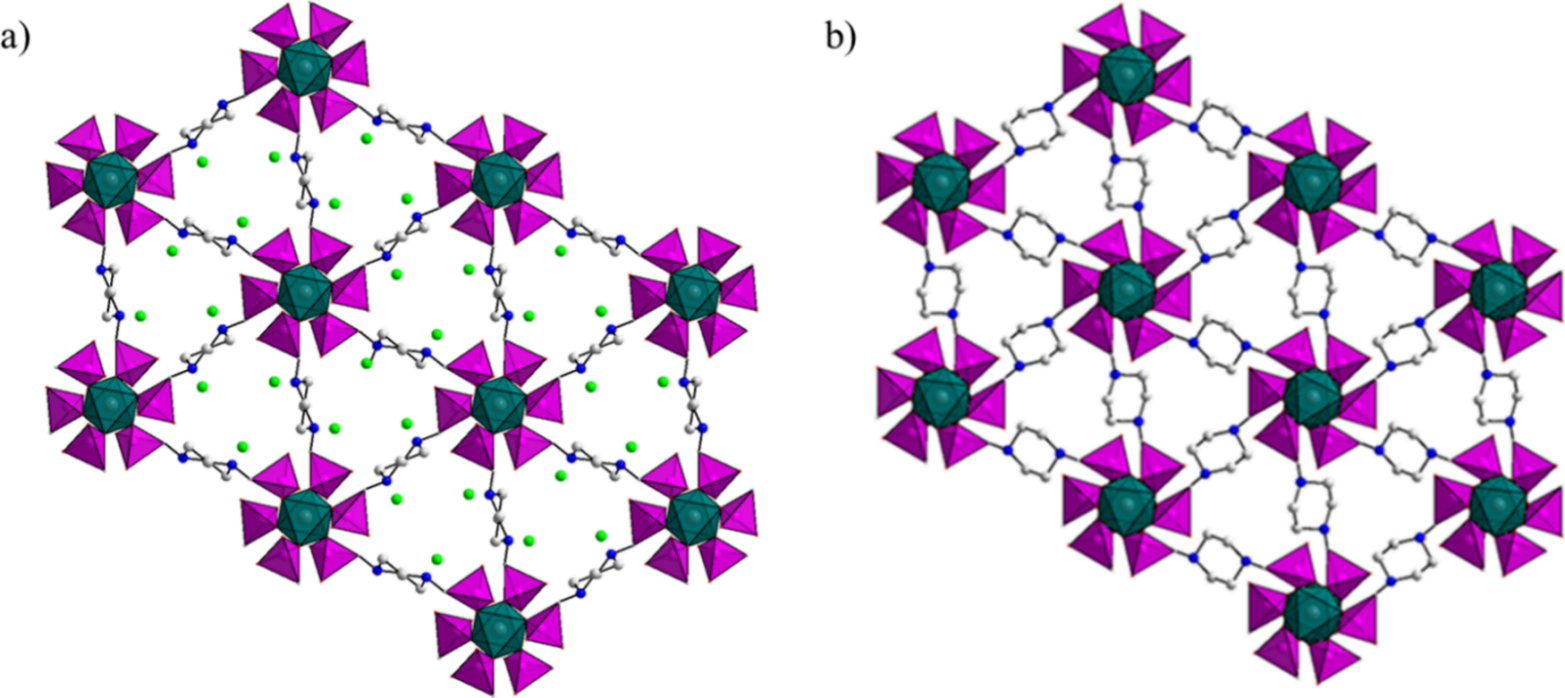
Structure of (a) Al-CAU-60·6HCl and (b) Al-CAU-60·9H_2_O. Aluminum is teal, oxygen is red, phosphorus is purple, carbon is gray, nitrogen is dark blue, and chlorine is green. Water molecules are omitted for clarity, polyhedral representation of the structures viewed along the *c* axis.

Thermal analysis further supported these findings. S1 and S3 samples displayed the typical two-step mass loss profile: the first corresponding to solvent release (H_2_O, DMF) below 200 °C, and the second to framework decomposition above 350 °C (Figure 8d and Figure S7). In contrast, the HCl-loaded sample (S2) exhibited an additional mass loss step between 225 and 350 °C, accounting for approximately 6.5 wt % (Figure 8d and Figure S10b). This intermediate weight loss is attributed to HCl-induced structural decomposition,^38^ which also explains the observed decline in adsorption capacity after thermal regeneration at 250 °C (Figure S10a). Consistent with this, the PXRD pattern of thermally regenerated pellets (Figure S11) showed a peak shift from 9.10° to 8.50°, indicating framework alteration due to residual HCl and confirming that thermal treatment at 250 °C was insufficient for full regeneration. Altogether, these findings confirm that the structured Al-CAU-60/PVF pellets preserve the fundamental adsorption-regeneration mechanism of the pristine MOF corresponding to protonation of the phosphonate groups during HCl adsorption and deprotonation during aqueous regeneration. Importantly, this reversible switching occurs without compromising the structural integrity or performance of the material.

### HCl Removal Performance Comparison

To assess the practical applicability of Al-CAU-60/PVF pellets for gas purification, their HCl removal performance was benchmarked against a widely used commercial adsorbent, zeolite 13X. Zeolites such as 13X are commonly employed in industrial gas purification due to their high surface area and proven adsorption capacity.^52^ However, their limited regenerability in HCl adsorption and reduced chemical stability under acidic conditions present operational challenges.^22^

In this section, a direct comparison of dynamic HCl adsorption behavior under identical experimental conditions is presented to assess the relative performance, reusability, and potential of Al-CAU-60/PVF pellets for industrial applications. To ensure comparability, 13X was processed using the same binder (PVF) and extrusion–crushing–sieving procedure applied for shaping the Al-CAU-60 pellets. PXRD analysis confirmed that the crystalline framework of 13X remained intact after pelletization (Figure S15). Subsequently, breakthrough experiments were conducted using packed beds of 13X/PVF pellets under identical dynamic conditions. In parallel, the regeneration potential of 13X/PVF pellets was examined using thermal treatment and water immersion, followed by a second HCl breakthrough test to assess performance recovery. Three consecutive adsorption-regeneration cycles were performed using the water-triggered route (see Section S7 of the Supporting Information for details). Figure 10 displays the resulting HCl breakthrough profiles along with the calculated adsorption capacities (at 1 ppm of HCl breakthrough), which are compared against those of Al-CAU-60/PVF pellets for the first three cycles. In the first cycle, 13X/PVF pellets displayed a breakthrough time of ∼25 min and a 70% higher breakthrough capacity (2.84 mmol/g) compared to Al-CAU-60/PVF. The breakthrough profile of 13X exhibited a sharp elution curve similar to that of Al-CAU-60 (Figure 10a and Figure S16), and reached saturation after approximately 90 min, corresponding to a capacity of 3.96 mmol/g. This performance is consistent with the literature.^22^

**Figure 10:**
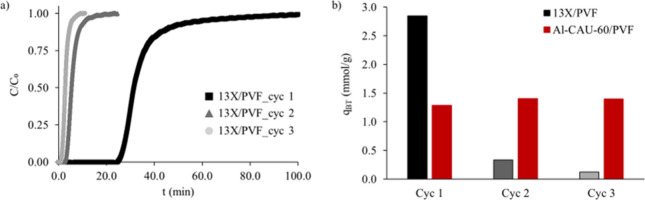
(a) HCl breakthrough curves measured using packed columns containing 13X/PVF pellets at 25 °C, 0.30 m/s gas velocity, 1.03 mbar HCl partial pressure, and 10 bar total pressure and (b) HCl adsorption capacities at the 1 ppm breakthrough point, calculated from the breakthrough curves for 13X/PVF and Al-CAU-60/PVF pellets.

The higher HCl uptake of 13X relative to Al-CAU-60 is attributed to its large surface area and high extra-framework cation density, which promote strong interactions with HCl molecules. The extra-framework Na^+^ cations in 13X serve as active sites for HCl chemisorption, forming NaCl via direct reaction.^22^ In contrast, HCl capture in Al-CAU-60 occurs via protonation of phosphonate groups present in the framework.^38^ Despite its initially higher capacity, 13X showed poor regenerability: after the first water-triggered cycle, its capacity dropped by ∼88%, further decreasing to ∼96% after the third. This decline aligns with PXRD results (Figure S15), which revealed hydrated salt formation within the zeolite pores after HCl exposure, seen as new peaks at 26.6°, 32.1°, and 42.4°.^22^ In contrast, Al-CAU-60/PVF retained stable performance across cycles, with an average breakthrough capacity of 1.38 mmol/g (Figure 10b). A thermal regeneration route was also tested by heating the packed column to 250 °C under He flow for 8 h, followed by a subsequent HCl adsorption test. Figure S17 shows the resulting breakthrough curve plotted as a function of time before and after thermal regeneration. A drop of around 90% in the breakthrough capacity (0.27 mmol/g) was noted, with a breakthrough time noted as 2.7 min. This sharp contrast highlights the superior cycling stability and water-triggered regenerability of Al-CAU-60/PVF under acidic conditions, establishing it as a promising candidate for industrial HCl removal.

## Conclusions

In this work, the aluminum-phosphonate MOF Al-CAU-60·9H_2_O was investigated for the removal of hydrogen chloride (HCl) from hydrogen gas streams via adsorption. A major outcome of this work is the successful scale-up of phosphonate-based Al-CAU-60·9H_2_O via a reproducible synthesis route under reflux conditions, achieving 10 L-scale production with high yield (>96%) and preserved crystallinity—overcoming one of the key barriers in phosphonate MOF synthesis. Following synthesis, Al-CAU-60 was shaped into mechanically robust pellets (Al-CAU-60/PVF) using 10.0 wt % polyvinyl formal (PVF) as a binder. Characterization confirmed retention of the MOF’s structural and chemical integrity, as shown by consistent PXRD patterns, ATR-FTIR spectra, and thermal stability in TGA analysis. Dynamic breakthrough experiments under practically relevant conditions demonstrated that Al-CAU-60/PVF pellets possess an average HCl adsorption capacity of 1.38 mmol_HCl_/g_Al‑CAU‑60/PVF_ at the 1 ppm breakthrough point, and 1.73 mmol_HCl_/g_Al‑CAU‑60/PVF_ at saturation. These performances were reproducible across independently synthesized batches, confirming the reliability of both the synthesis and pelletization processes.

The second key achievement is the demonstration of efficient water-triggered regeneration, allowing complete recovery of HCl adsorption capacity through simple rinsing and reuse over seven consecutive cycles. Al-CAU-60/PVF retained ∼89% of the HCl adsorption capacity measured for the pristine, binder-free MOF, while showing stable performance over multiple cycles. The HCl uptake mechanism, consistent with that of the pristine MOF, involved reversible protonation of the phosphonate groups within the framework during adsorption, and deprotonation during aqueous regeneration. This mechanism was corroborated by SEM-EDX, PXRD, ATR-FTIR, and TGA analyses. A comparative assessment with zeolite 13X further revealed its rapid deactivation upon regeneration, thereby emphasizing the superior robustness of Al-CAU-60 under repeated cycling. Overall, this study demonstrates both the scalable synthesis of a phosphonate-based MOF and its exceptional cyclic performance enabled by water-triggered regeneration, establishing Al-CAU-60/PVF as a practical and sustainable solution for HCl removal in industrial gas purification.

## Supplementary Material



## Notes

Literature examples for materials used for hydrogen purification, synthesis optimization, PXRD data, BT measurement data, SEM micrographs, EDX, NMR, and IR spectroscopy data, and thermogravimetric analysis data (PDF)
