# Supplementary material for: Structured Aluminum Phosphonate Metal–Organic Framework for Efficient Hydrogen Purification: Reversible HCl Adsorption and Water-Triggered Regeneration
Source: Chem Mater. 2026 Apr 10;38(14):7083–94. doi: 10.1021/acs.chemmater.5c03397 (PMC13435743; doi:10.1021/acs.chemmater.5c03397)
Supplement: Supplementary file 1 [file cm5c03397_si_001.pdf]

Supporting information

# Structured Aluminum Phosphonate Metal-Organic Framework for Efficient Hydrogen Purification: Reversible HCl Adsorption and Water-Triggered Regeneration

*Ravi Sharma,[a] Ajay Padunnappattu,[a,b] Max Phil Van der Stricht,[a] Norbert Stock,[*b c] and Joeri F.M. Denayer [*a]*

[a] Chemical Engineering Department, Vrije Universiteit Brussel, B-1050, Belgium

[b] Institut für Anorganische Chemie, Christian-Albrechts-Universität zu Kiel, Max-Eyth-Str. 2, 24118 Kiel, Germany.

[c] Kiel Nano, Surface and Interface Science KiNSIS, Christian-Albrechts-Universität zu Kiel, Christian-Albrechts-Platz 4, 24118 Kiel, Germany.

## Table of Content

S1. Introduction 3

S2. Material synthesis 4

S2.1. Chemicals 4

S2.2. Synthesis of ligand 4

S2.3. Synthesis of Al-CAU-60·9H2O from Al-CAU-60·6HCl 5

S3. Al-CAU-60 synthesis: Scale-up 6

S3.1. Characterization 6

S3.2. Scanning Electron Microscopy (SEM) 7

S3.3. Crush Strength Test 9

S3.4. Energy Dispersive X-ray (EDX) analysis 10

S3.5. Fourier Transform – InfraRed (FT-IR) analysis 10

S3.6. Thermogravimetric Analysis (TGA) 11

S4. Reproducibility of the structuring method 12

S5. Reproducibility of the breakthrough experimentation 12

S6. HCl Adsorption and Regeneration by Thermal and Water-Triggered route 14

S7. HCl removal performance comparison with commercial adsorbent, 13X 16

References 18

## S1. Introduction

Table S1. Overview of sorbents employed for HCl removal

| Sorbents | HCl capacity (g/g)/ HCl inlet conc. | Regenerative | Ref. |
|---|---|---|---|
| $Al_2O_3.xH_2O$ | 0.07 / 5 ppm | No | [1] |
| $Al_2O_3$+Y zeolite+$Na_2O$ | 0.0038g/gh[a]/10 ppm | No | [2] |
| $Al_2O_3$+5-15wt.% $Na_2O$ | 0.18 / 1000 ppm | No | [3] |
| $Mg(OH)_2$+$Ca(OH)_2$ (15:85 mol %) | 0.93 / 13 % HCl | No | [4] |
| MgO- montmorillonite (30:70 wt. %) | 0.48 / 1 ppm | No | [5] |
| Mg-Al layered hydroxide/$\mathbf{CO_3^{2-}}$ | 0.0004 / 50 wt. % | Thermal, 250 °C | [6] |
| $K_2CO_3/Al_2O_3$ | 0.30 / 525 ppmv | No | [7] |
| 13X | 0.17 / 300 ppm | No | [8] |
| Zn-exchanged 13X | 0.20 / 300 ppm | No | [8] |
| UiO-66 (Zr) | 0.27 / 10 % | Thermal, 200 °C | [9] |
| MIL-101 (Cr) | 0.28 / 10 % | Thermal, 200 °C | [9] |
| Cr-MIL-101-$NH_2$ | 0.23 / 1000 ppm | Thermal, 150 °C | [10] |
| ZIF-8 | 1.02 / 200 ppm | No | [11] |
| HPU-23[c] | Detector 10 – 70 ppm | Yes, alkali gas | [12] |
| Al-CAU-60 | 0.18 / 1 mol/L[b] | Yes, water | [13] |

[a] HCl adsorption rate (10 ppm $HCl/N_2$, 50 cc/min, 25 °C)
[b] Aqueous HCl: $c \geq 1$ mol $L^{-1}$
[c] Porous fluorescent MOF

## S2. Material synthesis

### S2.1 Chemicals

$AlCl_3 \cdot 6H_2O$ (99 %, Sigma Aldrich), anhydrous piperazine (98 %, TCI chemicals), Formaldehyde (37 wt.% in $H_2O$, Sigma Aldrich), Phosphorus acid (99 %, Sigma Aldrich), hydrochloric acid (37 wt. %, pure, VWR) were used without further purification.

### S2.2 Synthesis of the ligand

The ligand N, N'-piperazinebis(methylenephosphonic acid) ($H_4PMP$) was synthesized following a reported procedure,[14] with modification introduced for large-scale synthesis. Piperazine (41.34 g, 0.48 mol) and phosphorus acid (102.36 g, 1.26 mol) were dissolved in a mixture of HCl (37 wt. %, 120 mL) and deionized water (120 mL). The solution was refluxed for 2 h, after which formaldehyde (120 mL, 37 wt. %) was added dropwise under continued stirring and heating. The reaction was maintained for 36 h. After cooling to room temperature, the volume of the mixture was reduced to 50 %. A 9:1 ethanol/water solution (200 mL) was added, and the mixture was stored overnight at 4 °C. The resulting solid was recovered by filtration, washed three times with 180 mL portions of 9:1 ethanol/water, and dried in an oven at 90 °C for 12 h, yielding 110 g of product (85% yield).

Table S2. Al-CAU-60·6HCl reaction conditions and yield for the synthesis in different volumes.

| Reaction Vessel | Reaction Time and Temperature | Reactant and molar ratio | Yield of reaction as compared to the metal % |
|---|---|---|---|
| 250 mL | 12 h, 125 °C[a] | 1.81 g (7.5 mmol) of $AlCl_3 \cdot 6H_2O$, 3.49 g (12.7 mmol) $H_4PMP$, 25 mL 37 wt. % HCl and 100 mL of water. | 4.45 g, 99.0 % |
| 500 mL | 12 h, 125 °C[a] | 3.62 g (15 mmol) of $AlCl_3 \cdot 6H_2O$, 6.98 g (25.5 mmol) $H_4PMP$, 50 mL HCl 37 wt. % and 200 mL of water. | 8.80 g, 98.0 % |
| 1 L | 12 h, 125 °C[a] | 7.24 g (30 mmol) of $AlCl_3 \cdot 6H_2O$, 13.96 g (50.9 mmol) $H_4PMP$, 100 mL 37 wt. % HCl and 400 mL of water. | 17.40 g, 97.4 % |
| 10 L | 14 h, 130 °C[a] | 62.4 g (0.26 mol) of $AlCl_3 \cdot 6H_2O$, 120 g (0.43 mol) $H_4PMP$, 787.5 mL 37 wt. % HCl and 3150 mL of water. | 151.50 g, 97.6 % |

[a] Temperature of the oil bath

### S2.3 Synthesis of Al-CAU-60·9$H_2O$ from Al-CAU-60·6HCl

The conversion of Al-CAU-60·6HCl to Al-CAU-60·9$H_2O$ was achieved by dispersing 10 g of Al-CAU-60·6HCl in 3 L of deionized water and stirred for 5 minutes, resulting in an initial suspension with pH ≈ 1. A NaOH (c = 2 mol/L) solution was then added dropwise under continuous stirring until the pH of the suspension reached 6. The mixture was further stirred for 30 minutes to ensure complete neutralization, after which the solid was recovered by vacuum filtration. The obtained solid was re-dispersed in 1 L of deionized water and stirred overnight, followed by filtration and washing with water (2 × 200 mL). The final product was dried in air to yield 8.63 g of Al-CAU-60 (~ 95 % yield). In this work, the term Al-CAU-60 is used to denote Al-CAU-60·9$H_2O$, unless stated otherwise.

## S3. Al-CAU-60 synthesis: Scale-up

### S3.1 Characterization

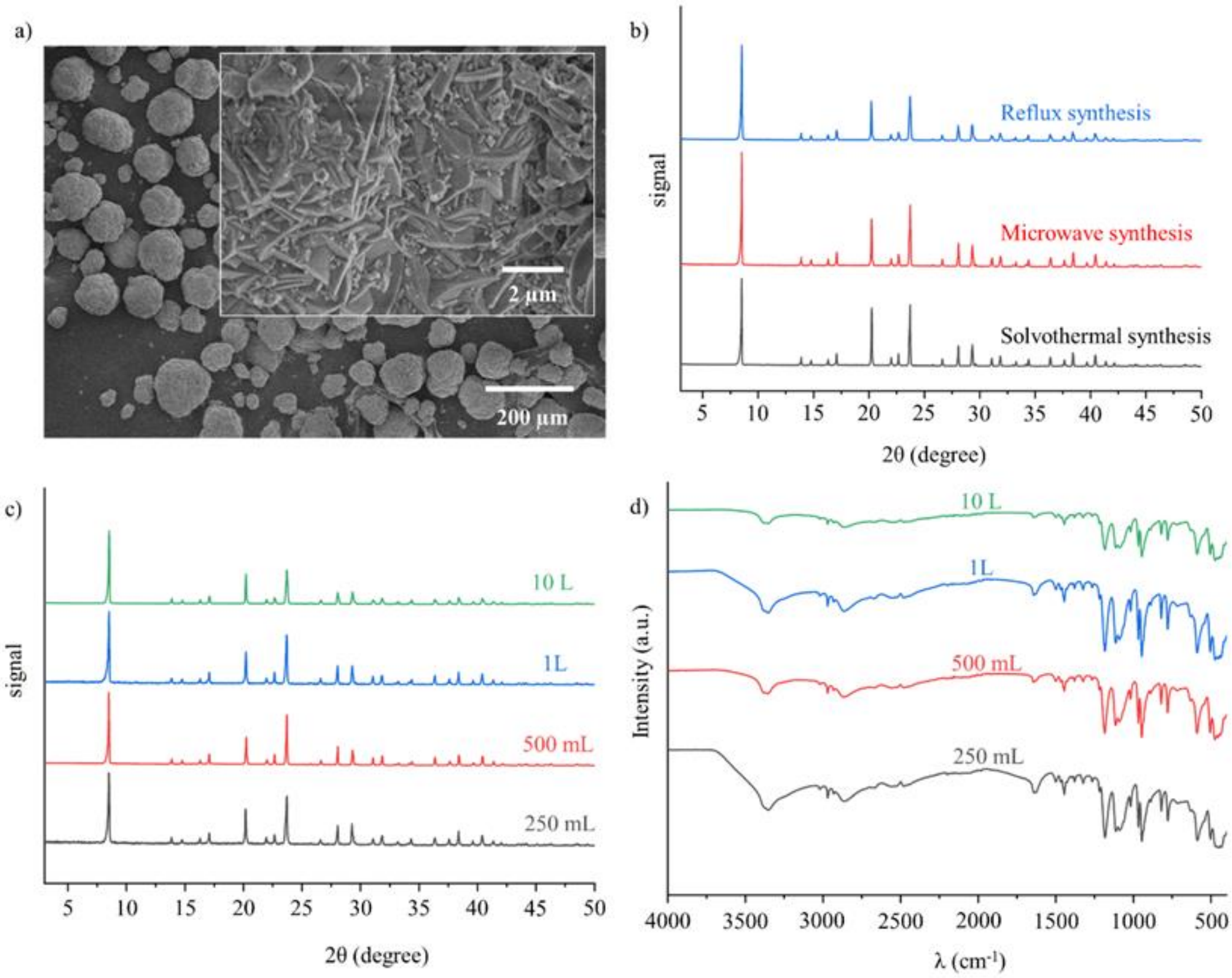


Figure S1. a) SEM micrograph of as-synthesized Al-CAU-60·6HCl; b) XRD patterns of Al-CAU-60·6HCl obtained from different synthesis routes, including conventional and microwave-assisted solvothermal, and reaction under reflux conditions; c) XRD patterns of Al-CAU-60·6HCl synthesized under reflux conditions at different batch scales (250 mL–10 L); and d) FTIR spectra of Al-CAU-60·6HCl synthesized under reflux conditions at different batch scales (250 mL–10 L).

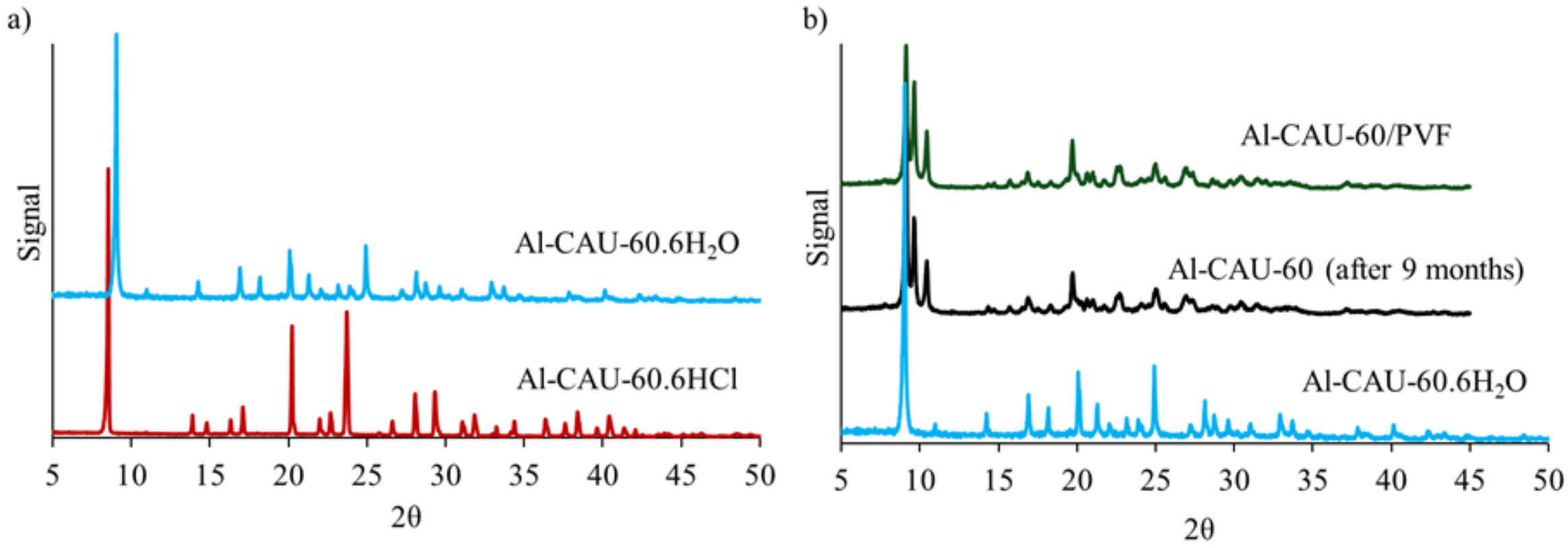


Figure S2. a) PXRD patterns of Al-CAU-60·6HCl and the freshly neutralized Al-CAU-60·$9H_2O$ after conversion, and (b) comparison with the XRD patterns of Al-CAU-60 after 9 months of storage and Al-CAU-60/PVF pellets. The term Al-CAU-60 is used throughout this work to denote Al-CAU-60·$9H_2O$ unless stated otherwise.

## S3.2 Scanning Electron Microscopy (SEM)

Figures S3 and S4 show the effect of binder content. Increasing the binder fraction resulted in well-preserved, mechanically robust pellets.

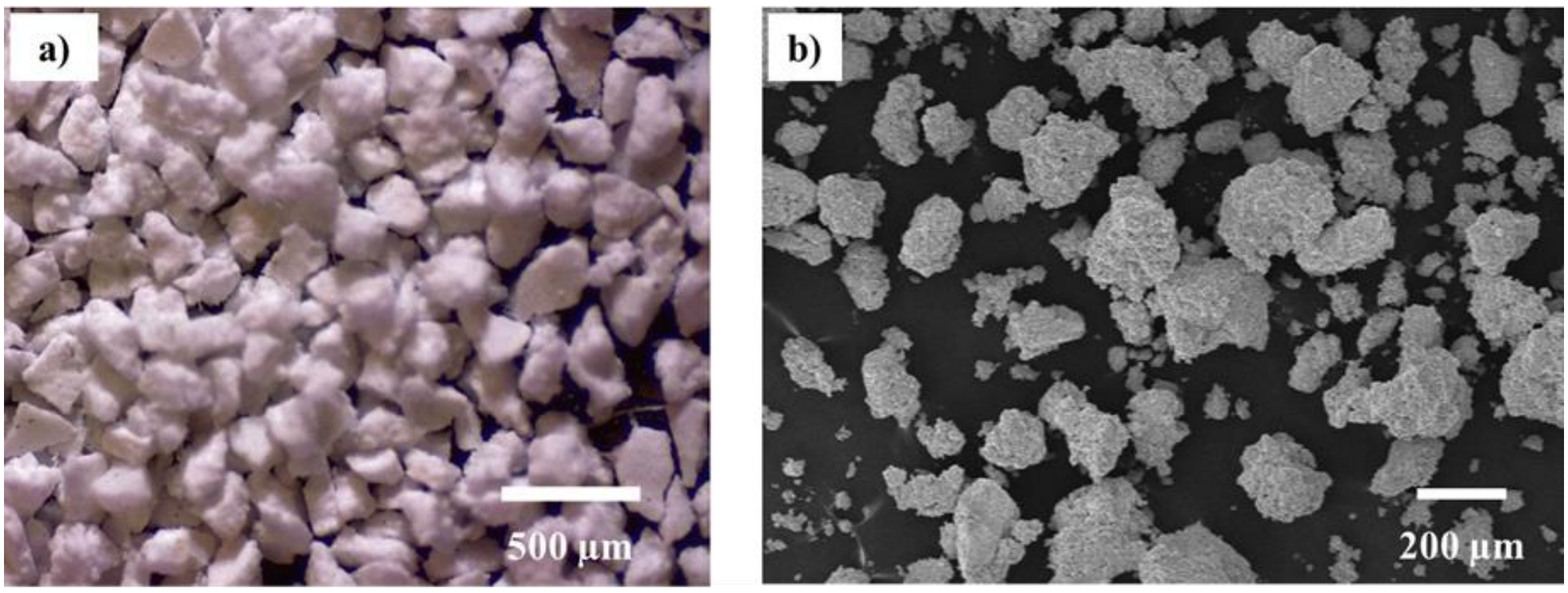


Figure S3. a) Optical micrograph of Al-CAU-60 pellets with 5.0 wt. % PVF and b) SEM micrograph of Al-CAU-60 pellets with 5.0 % PVF binder.

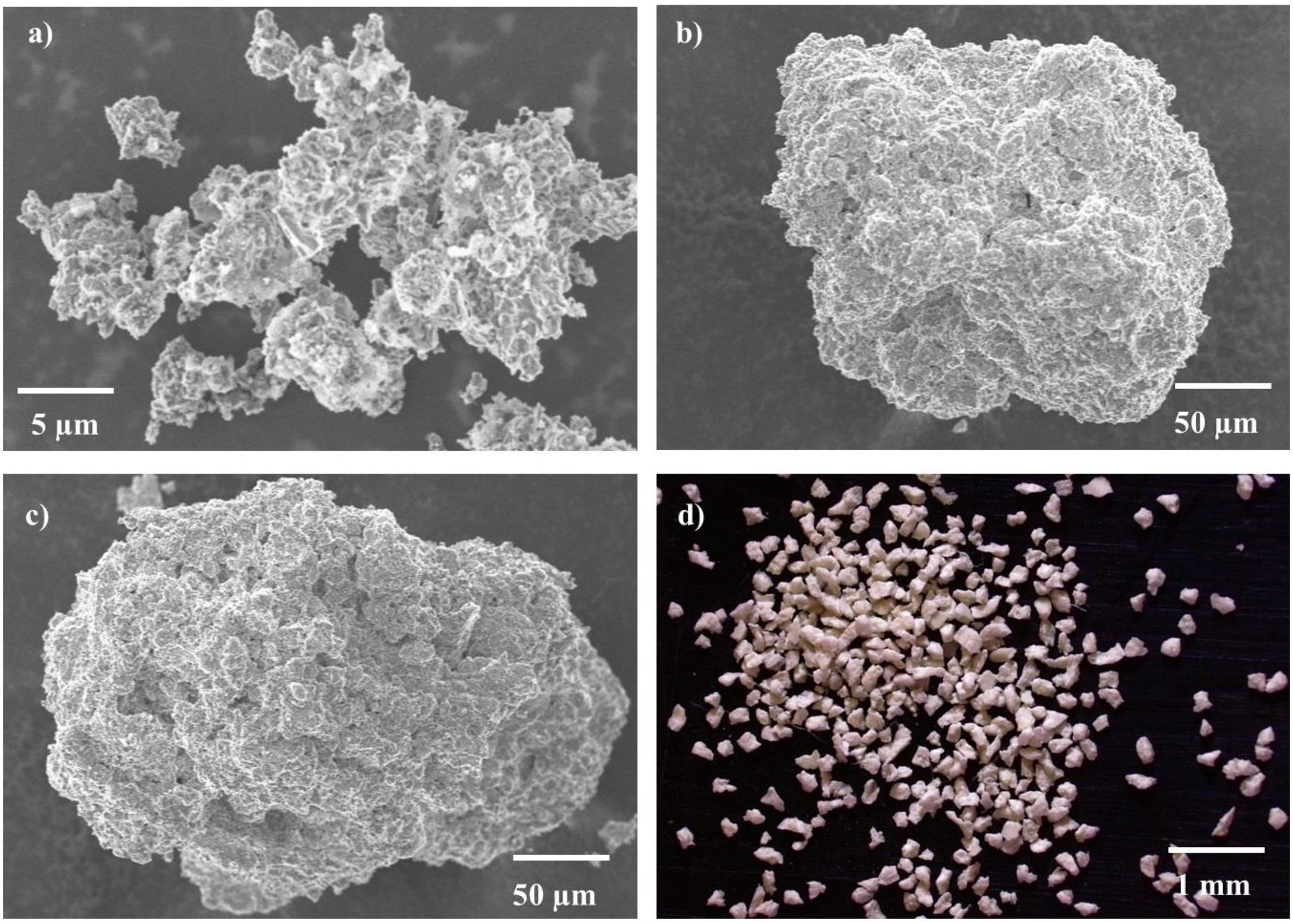


Figure S4. SEM micrographs of Al-CAU-60 (a) in pristine powder form, (b) in the pellet form with 10 wt. % binder (as-synthesized), (c) after five cycles of water immersion test and (d) optical micrograph of Al-CAU-60 pellets after five cycles of water immersion test. See section 2.4 of manuscript for details on water immersion test.

## S3.3 Crush Strength Test

Table S3. Crush strength test comparison with Al-CAU-60/PVF with other commercially available zeolites and MOFs

| **Sample Name** | **Supplier** | **Type** | **Form** | **Size (mm)** | **Crushing Strength (N)** | **Ref.** |
|---|---|---|---|---|---|---|
| 5A | Zhengzhou Gold Mountain Science and Technique Co. Ltd. | Zeolite | Bead | 2.0 - 2.8 | 30 | [15] |
| 5A | Zhengzhou Gold Mountain Science and Technique Co. Ltd. | Zeolite | Pellet | 1.6 | 18 | [15] |
| CMS-200 | Shanghai Jiuzhou Chemicals Co. Ltd | Carbon Molecular Sieve | Pellet | 1.1 - 1.2 | > 60/Pc | [15] |
| BPL4X10 | Chemviron | Actrivated Carbon | Granules | 1.0 - 5.0 | 14.2 | [15] |
| 13X-PG-1625 | Pukon United Group Co,. Limited | Zeolite | Bead | 1.6 - 2.5 | > 25 | [15] |
| ZIF-8/PVF | Cousin et.al. | MOF | Bead | 1.5 - 2.0 | 3.1 | [15] |
| 13X/PVF | this works | Zeolite | Extrudate | 2.0 - 2.2 | 66.6 | **this work** |
| Al-CAU-60/PVF | this work | MOF | Extrudate | 2.0 - 2.2 | 20.3 | **this work** |
| 13X/PVF | this works | Zeolite | Pellet | 1.0 - 1.1 | 9.8 | **this work** |
| Al-CAU-60/PVF | this work | MOF | Pellet | 1.0 - 1.1 | 4.5 | **this work** |

### S3.4 Energy Dispersive X-ray (EDX) analysis

Three batches of Al-CAU-60/PVF pellets were prepared following the methodology described in the section 2.3 of the manuscript. The pellets were polished to expose the cross-section and then coated with silver prior to EDX analysis.[16,17] The binder content was determined from the change in aluminum weight percentage before and after pelletization, assuming proportional dilution by the binder, [15,16] and was found to be 10.8, 11.6, and 12.8 wt. %.

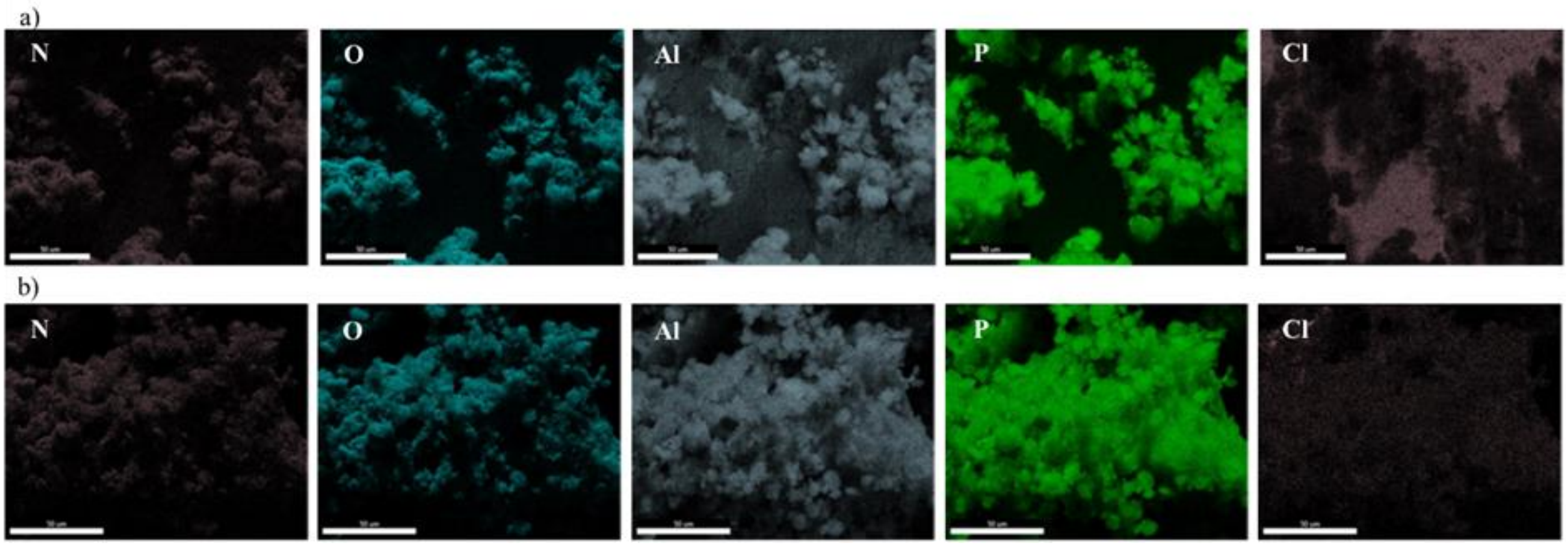


Figure S5. Elemental mapping of a) Al-CAU-60 crystals and b) Al-CAU-60/PVF pellets (Batch 2). (Scale: 50 µm).

### S3.5 Fourier Transform – InfraRed (FT-IR) analysis

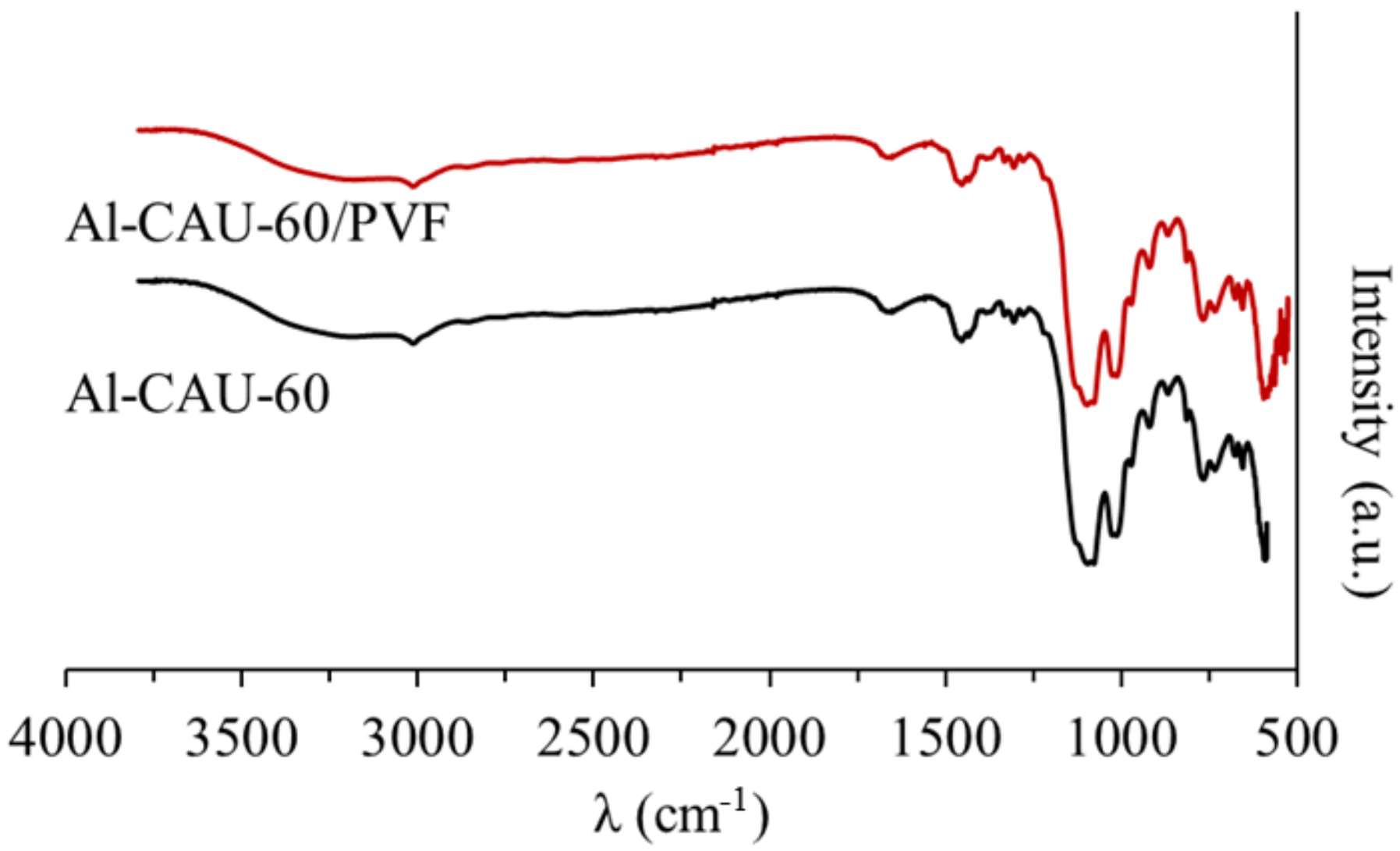


Figure S6. Attenuated total reflectance – Fourier transform infrared (ATR-FTIR) spectra of Al-CAU-60 crystals and Al-CAU-60/PVF pellets (mixed batch).

Figure S6 demonstrates that the chemical structure of Al-CAU-60 is retained in the pellet form.

## S3.6 Thermogravimetric analysis (TGA)

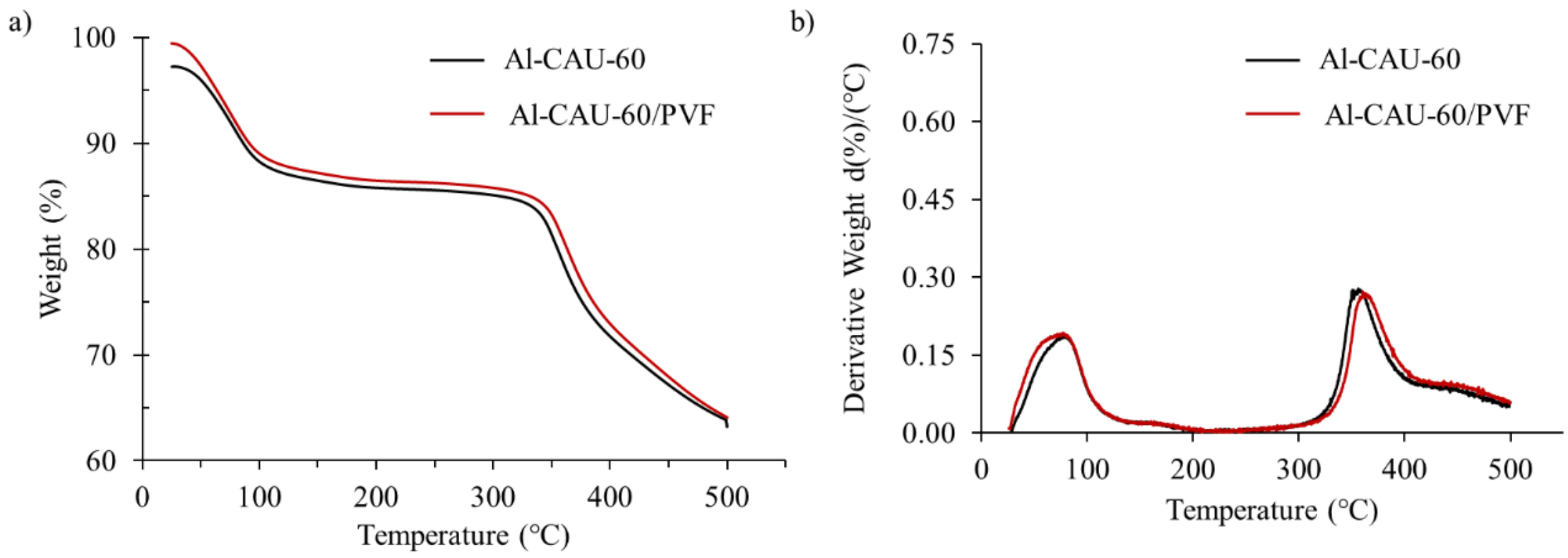


Figure S7. Thermogravimetric analysis (TGA) of Al-CAU-60 crystals and Al-CAU-60/PVF pellets (mixed batch) a) weight change and b) derivative weight change, showing two step mass loss: the first corresponding to solvent release ($H_2O$, DMF) below 200 °C, and the second to framework decomposition above 350 °C.

The TGA profiles of pristine Al-CAU-60 and Al-CAU-60/PVF pellets are identical.

Table S4. Mass of Al-CAU-60/PVF pellets after each cycle of water immersion test.

| Initial mass (g) | Cyc 1 | Cyc 2 | Cyc 3 | Cyc 4 | Cyc 5 |
|---|---|---|---|---|---|
| 103.42 | 102.87 | 102.27 | 101.52 | 100.76 | 100.23 |

## S4. Reproducibility of the structuring method

The reproducibility of the structuring method was evaluated by preparing three batches under identical conditions i.e., mixing Al-CAU-60 crystal powder with a PVF/DMF binder solution until obtaining a thick slurry, which later was extruded, dried, crushed upon drying and sieved. The binder fraction was 10.0 wt. %. The pellets obtained from these batches were characterized by EDX analysis and breakthrough experiments (Table S4 and Figure S2).

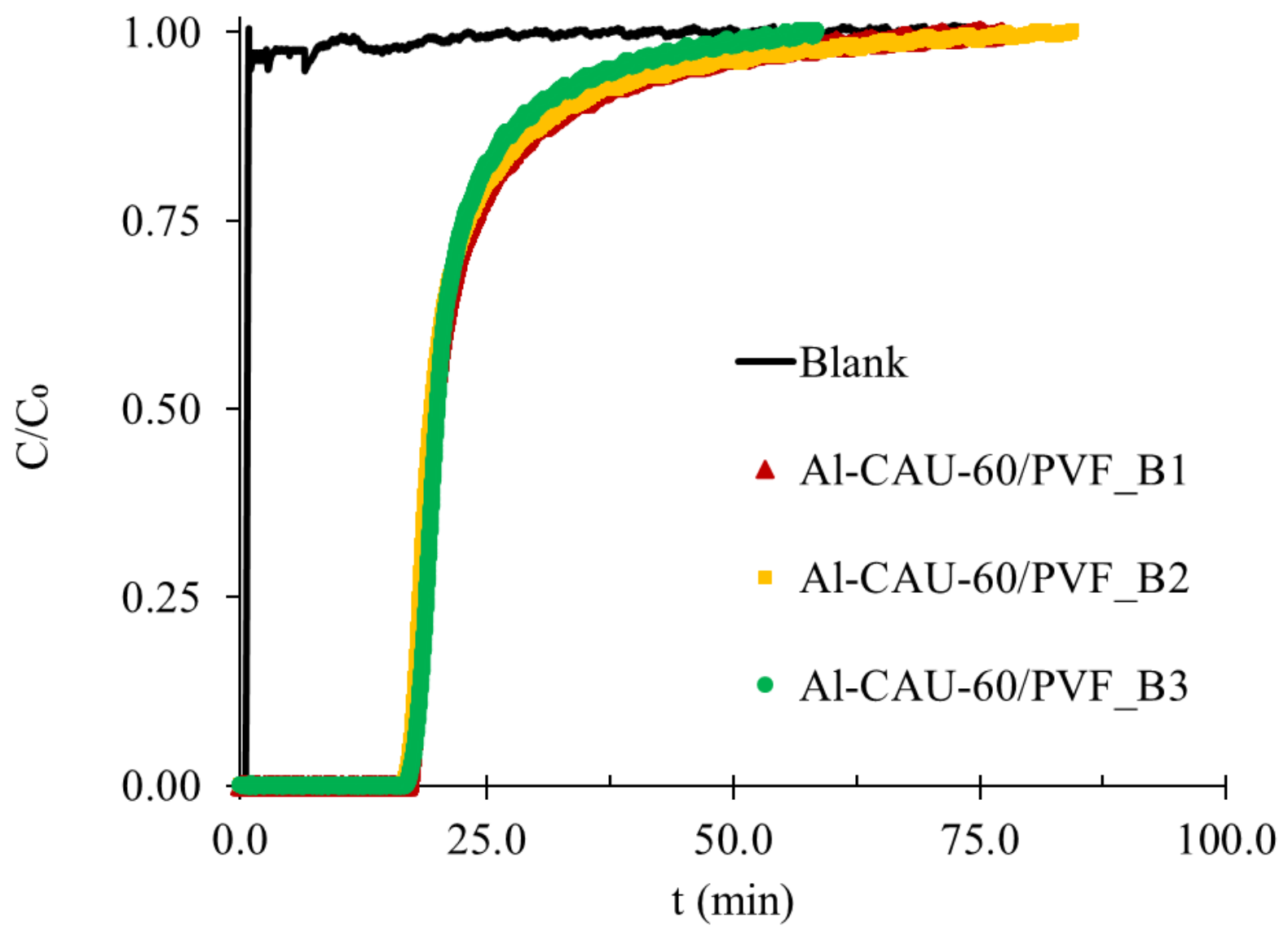


Figure S8. HCl breakthrough curves measured with packed columns containing Al-CAU-60/PVF pellets, at 25 °C, 0.30 m/s gas velocity, 1.03 mbar HCl partial pressure and 10 bar total pressure. Three batches were measured.

## S5. Reproducibility of the breakthrough experimentation

The reproducibility of the HCl breakthrough curves was verified with three measurements performed under identical conditions using pristine, binderless Al-CAU-60 pellets. Additional tests were conducted by varying the column length/loading, expressed as the mass of pellets placed inside the column (Table S4). From the resulting elution profiles, the corresponding amount of adsorbed HCl was calculated (Table S4). More details about the experimentation procedure are provided in the manuscript. The results clearly demonstrate the reproducibility of the breakthrough method. Identical performance was observed for three batches of Al-CAU-60/PVF pellets and across different column lengths (Table S5).

Table S5. Adsorption and chemical properties of Al-CAU-60/PVF batches

| Batch | Adsorbent mass (mg) | Binder content [a] | Binder content [b] | $q_{HCl,1ppm}$ (mmol/g) |
|---|---|---|---|---|
| Binderless | 65.0 | - | - | 1.45 |
| 1 | 78.0 | 11.6 | 10.3 | 1.30 |
| 2 | 75.0 | 10.8 | 11.0 | 1.29 |
| 3 | 76.0 | 12.8 | 12.4 | 1.27 |
| 2 | 40.0 | 10.8 | 11.0 | 1.29 |
| 3 | 22.0 | 12.8 | 12.4 | 1.27 |

a: Calculated from EDX analysis
b: Calculated from adsorption capacity

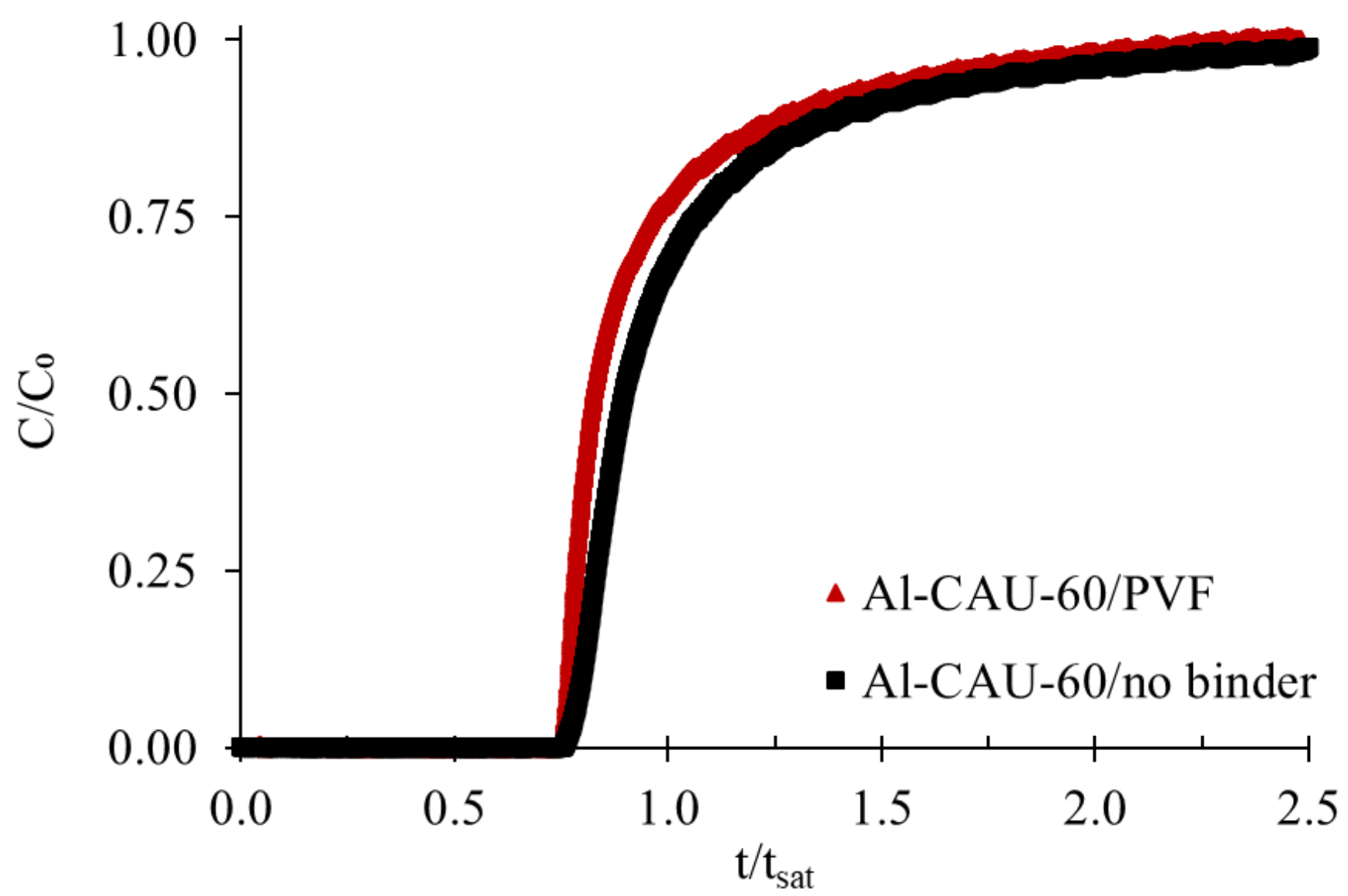


Figure S9. HCl breakthrough profiles for beds packed with binderless Al-CAU-60 pellets and Al-CAU-60/PVF pellets (200 – 280 µm) as a function of dimensionless time defined as ratio of operating time (t) to stoichiometric (average) breakthrough time ($t_{sat}$) obtained at 25 °C, 0.30 m/s gas velocity, 1.03 mbar HCl partial pressure and 10 bar total pressure.

## S6. HCl Adsorption and Regeneration by Thermal and Water-Triggered route

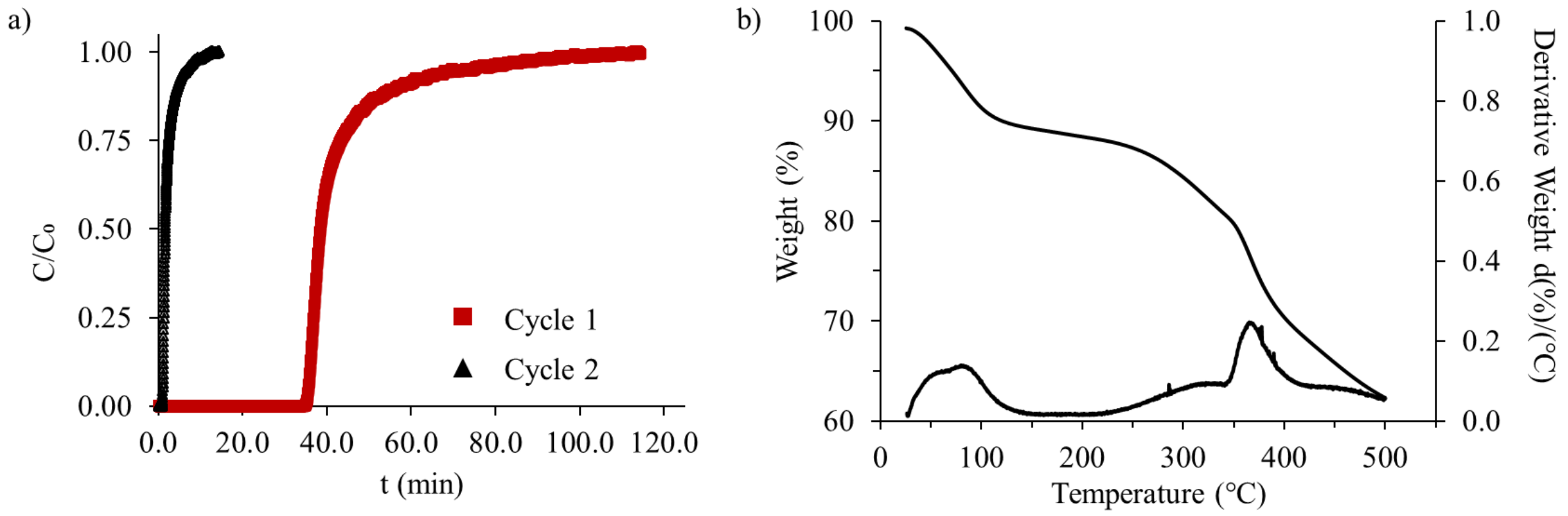


Figure S10. a) HCl breakthrough profiles for beds packed with Al-CAU-60/PVF pellets before (Cycle 1) and after thermal regeneration performed at 250 °C (Cycle 2). Both breakthrough experiments were performed at 25 °C, 0.30 m/s gas velocity, 1.03 mbar HCl partial pressure and 10 bar total pressure and b) Thermogravimetric analysis (TGA) of Al-CAU-60/PVF pellets after HCl adsorption along with derivative weight.

The thermal regeneration route was found to be ineffective and TGA analysis revealed an additional mass loss between 225 and 350 °C.

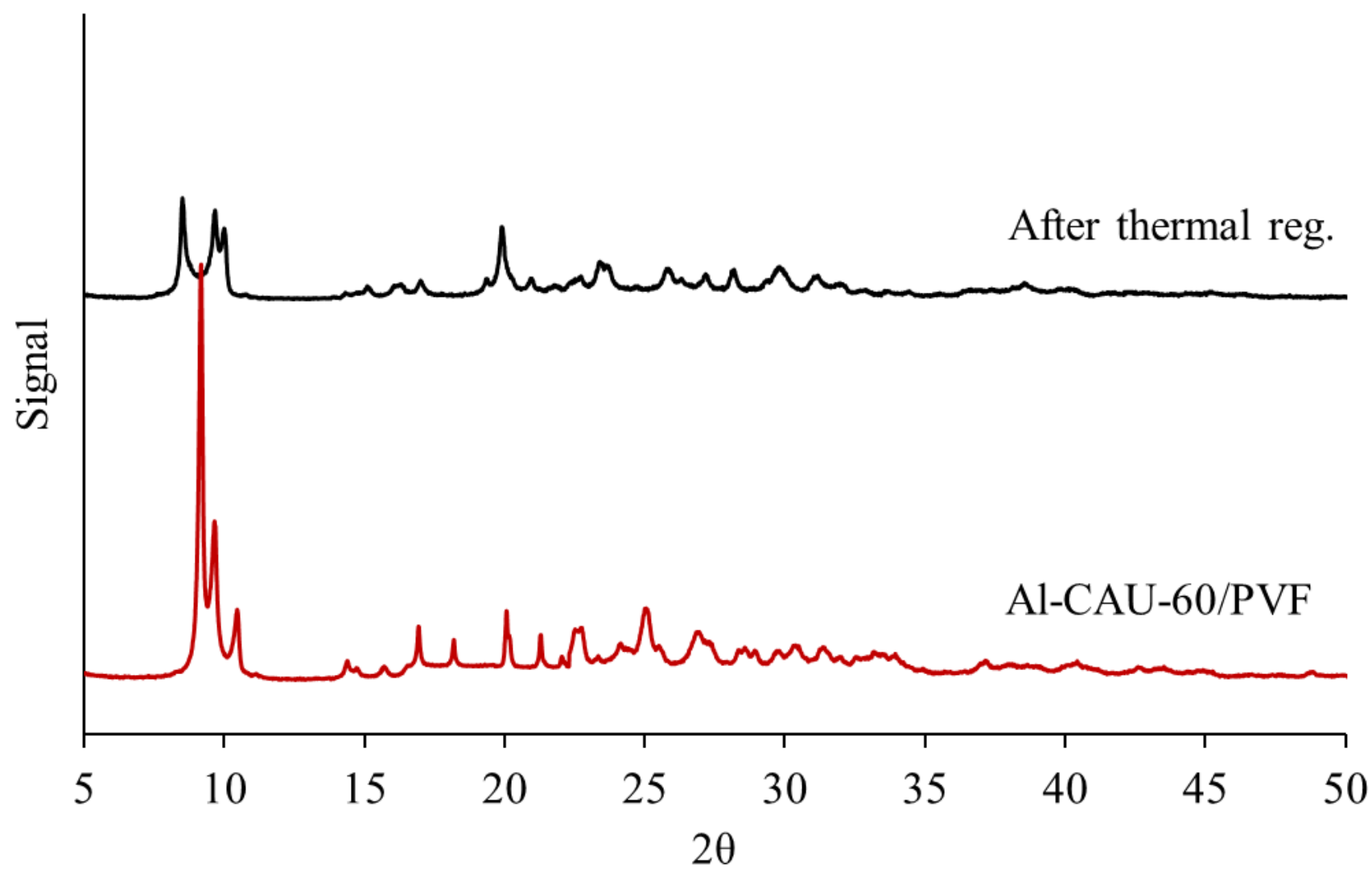


Figure S11. PXRD pattern of Al-CAU-60/PVF pellets before HCl adsorption (below, red) and HCl saturated Al-CAU-60/PVF pellets after thermal regeneration at 250 °C.

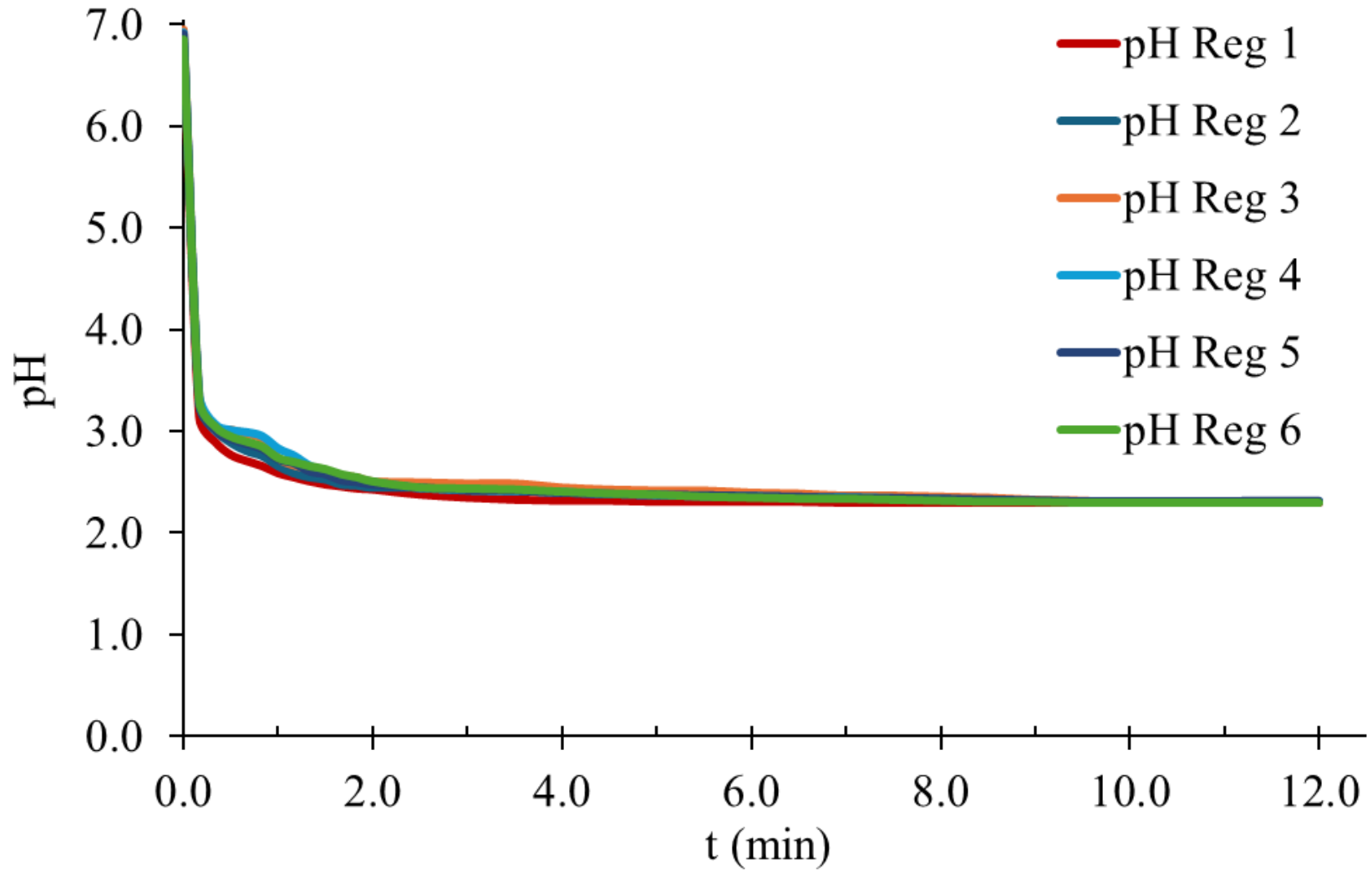


Figure S12. Time-dependent pH measurements during the dispersion of Al-CAU-60/PVF pellets in deionized water over multiple regeneration cycles.

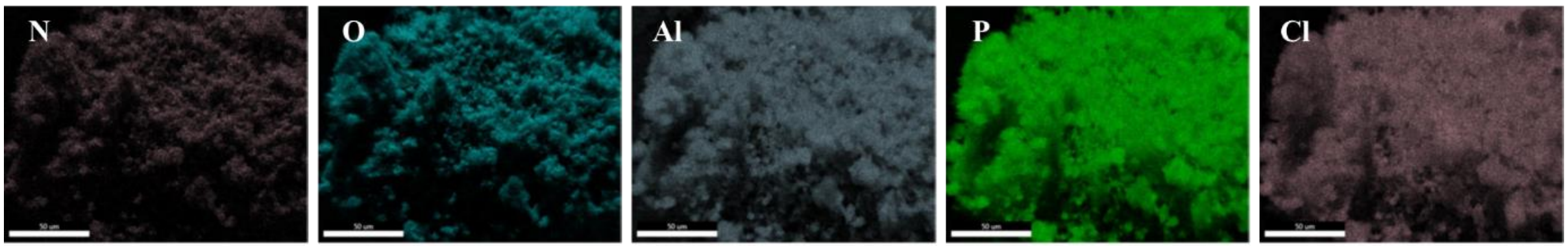


Figure S13. Elemental mapping of Al-CAU-60/PVF pellets after HCl adsorption (Scale: 50 μm).

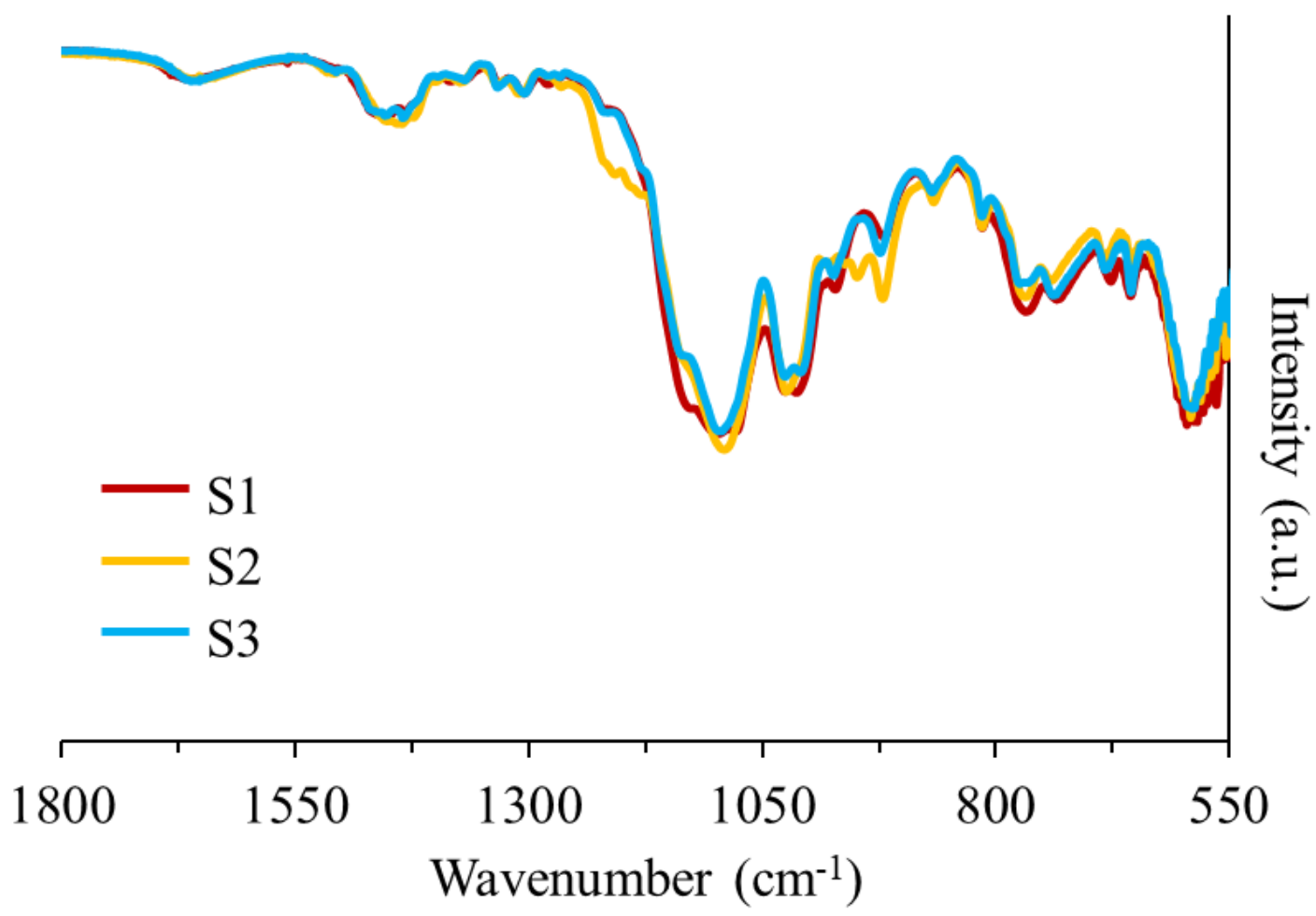


Figure S14. Attenuated total reflectance – Fourier transform infrared (ATR-FTIR) spectra analyses of Al-CAU-60/PVF pellets before HCl contact (S1), after HCl exposure (S2) and after water-triggered desorption (S3).

## S7. HCl removal performance comparison with commercial adsorbent, 13X

The 13X/PVF pellets were synthesized following the same methodology described for Al-CAU-60. In brief, 1.0 g of 13X zeolite powder (UOP) was mixed with a PVF binder solution (10.0 wt.%) in DMF solvent until a homogeneous thick slurry was obtained. The resulting slurry was then extruded and left to dry overnight in open air. Once dried, the extrudates were crushed and sieved to obtain particles within size range of 200 – 280 µm. Figure S15 shows the PXRD pattern of pristine zeolite 13X crystals along with zeolite 13X/PVF pellets.

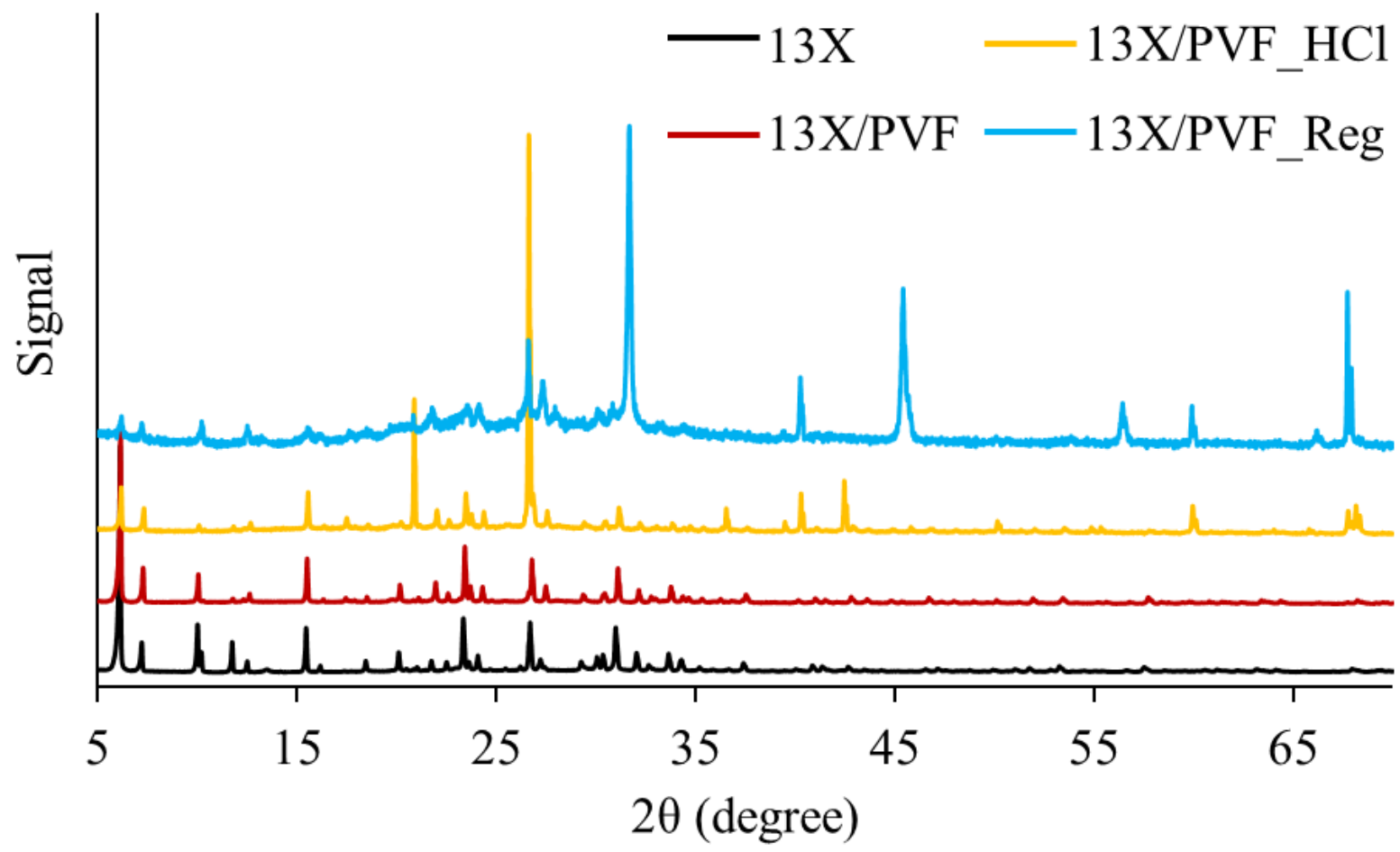


Figure S15. PXRD patterns of pristine zeolite 13X (bottom, black), zeolite 13X/PVF pellets before HCl exposure (second from bottom, red), after HCl exposure (second from top, yellow), and after water-triggered regeneration (top, blue).

The zeolite 13X pellets were packed in a column of length 5 cm and inner diameter 0.216 cm and subjected to breakthrough experiments at 25 °C, 0.30 m/s gas velocity, 1.03 mbar HCl partial pressure, and 10 bar total pressure. Figure S16 exhibits the breakthrough curve plotted as a function of dimensionless time, i.e., the ratio of operating time, t, to stoichiometric breakthrough time, $t_{sat}$ along with HCl adsorption capacities at saturation and at 1 ppm breakthrough point. The zeolite 13X/PVF pellets after HCl adsorption were regenerated via two routes: thermally at 250 °C and water-triggered route. For thermal route, the column was heating under He flow at 1 °C/min until 250 °C and maintained for 8h. For the water-triggered route, the pellets were submerged in deionised water for 1 h and later recovered via filtration and subjected to drying at 70 °C for 2 h. Figure S15 depicts the PXRD pattern of 13X/PVF pellets after HCl contact and after water-triggered regeneration. The treated pellets were then subjected to a second HCl breakthrough experiment under the same conditions as the initial test (*vide supra*).

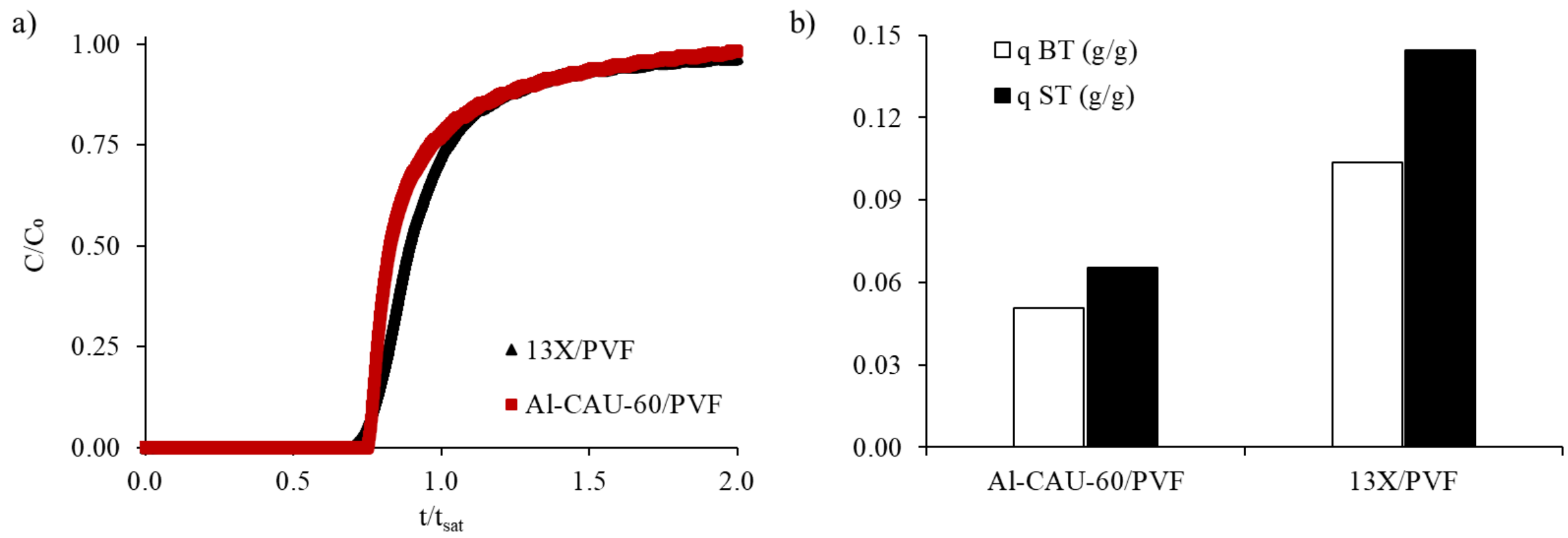


Figure S16. a) Breakthrough profiles of cycle 1 plotted as a function of dimensionless time, defined as ratio of operating time (t) to stoichiometric (average) breakthrough time ($t_{sat}$) for 13X/PVF and Al-CAU-60/PVF pellets, and b) HCl adsorption capacities at saturation (filled bars, ST) and at 1 ppm breakthrough point (empty bars, BT), obtained from break through curves for 13X/PVF and Al-CAU-60/PVF pellets. Breakthrough curves were recorded at 25 °C, 0.30m/s gas velocity, 1.03 mbar HCl partial pressure and 10 bar total pressure.

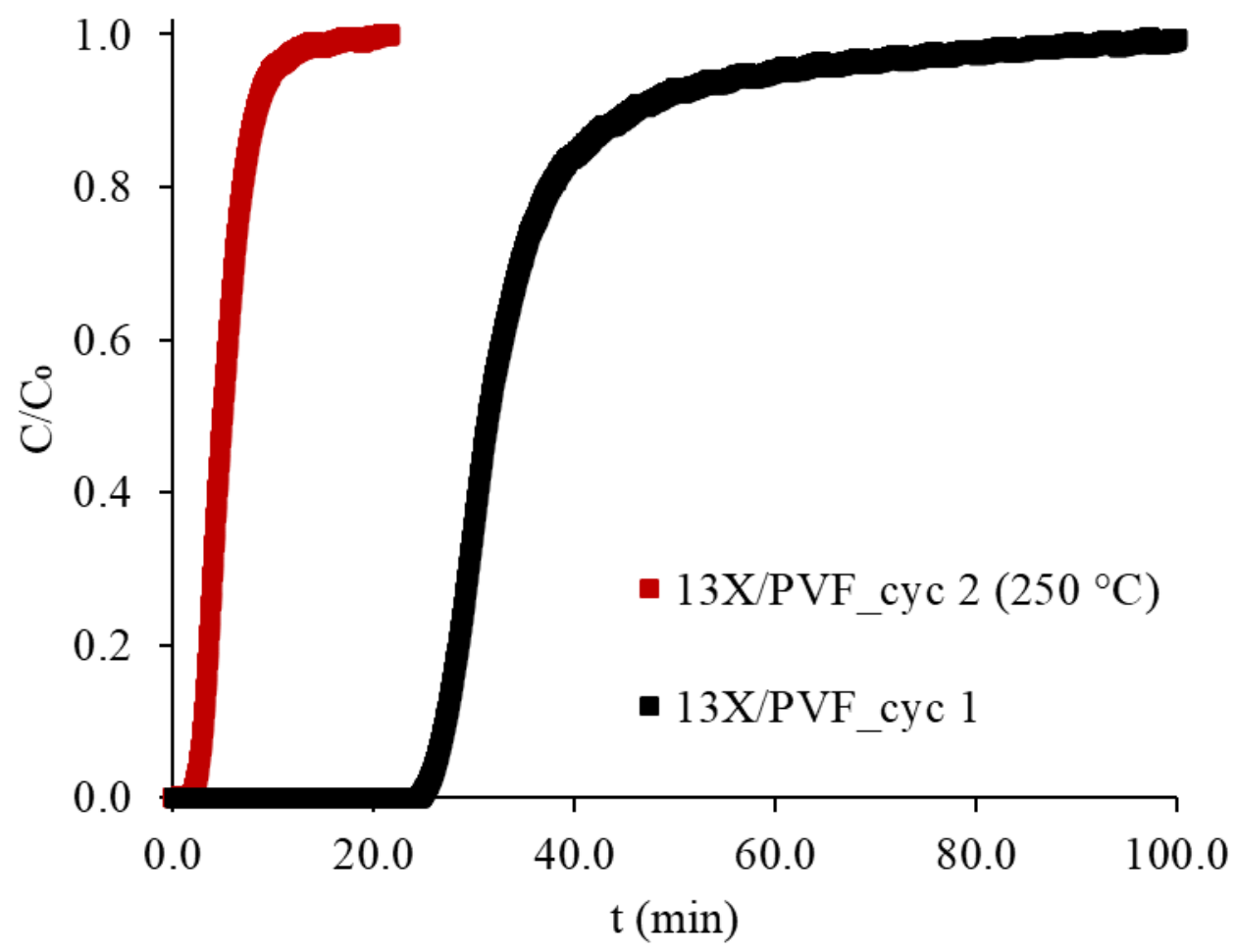


Figure S17. Breakthrough profile 13X/PVF pellets before (13/PVF_cyc 1) and after thermal regeneration (13X/PVF_cyc 2 (250 °C)). The curves were recorded at 25 °C, 0.30m/s gas velocity, 1.03 mbar HCl partial pressure and 10 bar total pressure.

## REFERENCES


(1) Pearson, M. J.; Promoted scavenger for purifying HCl-contaminated gases, **1987**.

(2) Fleming, H. L.; Goodboy K. P.; Saforo E. K., Adsorbent for HCl comprising alumina and acid-treated Y zeolite, **1988**.

(3) Lee J. S.; Pearson M. J., HCl adsorbent and method for making and using same, **1994**.

(4) Chao C. C., Process for removing HCl from hydrocarbon streams, **1997**.

(5) Dou, B.; Pan, W.; Ren, J.; Chen, B.; Hwang, J.; Yu, T. U., Single and combined removal of HCl and alkali metal vapor from High-Temperature Gas by Solid Sorbents. *Energy and Fuels* **2007**, *21* (2), 1019–1023. DOI: 10.1021/ef060266c.

(6) Kameda, T.; Uchiyama, N.; Park, K. S.; Grause, G.; Yoshioka, T., Removal of Hydrogen Chloride from Gaseous Streams Using Magnesium-Aluminum Oxide. *Chemosphere* **2008**, *73* (5), 844–847. DOI: 10.1016/j.chemosphere.2008.06.022.

(7) Kim, J. Y.; Park, Y. C.; Jo, S. H.; Ryu, H. J.; Baek, J. I.; Moon, J. H., The Absorption Breakthrough Characteristics of Hydrogen Chloride Gas Mixture on Potassium-Based Solid Sorbent at High Temperature and High Pressure. *Energy and Fuels* **2016**, *30* (3), 2268–2275. DOI: 10.1021/acs.energyfuels.5b02473.

(8) Sharma, R.; Segato, T.; Delplancke, M. P.; Terryn, H.; Baron, G. V.; Denayer, J. F. M.; Cousin-Saint-Remi, J. Hydrogen Chloride Removal from Hydrogen Gas by Adsorption on Hydrated Ion-Exchanged Zeolites. *Chem. Eng. J.* **2020**, *381*. DOI: 10.1016/j.cej.2019.122512.

(9) Liu, J.; Xia, W.; Mu, W.; Li, P.; Zhao, Y.; Zou, R. New Challenge of Metal-Organic Frameworks for High-Efficient Separation of Hydrogen Chloride toward Clean Hydrogen Energy. *J. Mater. Chem.* **2015**, *3* (10), 5275–5279. DOI: 10.1039/C4TA06832G.

(10) Gargiulo, N.; Peluso, A.; Aprea, P.; Micoli, L.; Ausiello, A.; Turco, M.; Marino, O.; Cioffi, R.; Jannelli, E.; Caputo, D. Use of a Metal Organic Framework for the Adsorptive Removal of Gaseous HCl: A New Approach for a Challenging Task. *ACS Appl. Mater. Interfaces* **2018**, *10* (17), 14271–14275. DOI: 10.1021/acsami.8b03007.

(11) Sharma, R.; Cousin-Saint-Remi, J.; Tiriana, S.; Delplancke, M. P.; Pletincx, S.; Baert, K.; Hauffman, T.; Terryn, H.; Baron, G. V.; Denayer, J. F. M. Metal-Organic Framework ZIF-8 for Exceptional HCl Removal from Hydrogen Gas by Reaction. *Int. J. Hydrogen Energy* **2022**, *47* (47), 20556–20560. DOI: 10.1016/j.ijhydene.2022.04.244.

(12) Xu, X.; Li, H.; Xu, Z. Multifunctional Luminescent Switch Based on a Porous PL-MOF for Sensitivity Recognition of HCl, Trace Water and Lead Ion. *Chem. Eng. J.* **2022**, *436*. DOI: 10.1016/j.cej.2022.135028.

(13) Reichenau, T. M.; Steinke, F.; Wharmby, M. T.; Näther, C.; Engesser, T. A.; Stock, N. Targeted Synthesis of a Highly Stable Aluminium Phosphonate Metal–Organic Framework Showing Reversible HCl Adsorption. *Angew. Chem. Int. Ed.* **2023**, *62* (26). DOI: 10.1002/anie.202303561.

(14) Benoit, V.; Pillai, R. S.; Orsi, A.; Normand, P.; Jobic, H.; Nouar, F.; Billemont, P.; Bloch, E.; Bourrelly, S.; Devic, T., et al. MIL-91(Ti), a Small Pore Metal-Organic Framework Which Fulfils Several Criteria: An Upscaled Green Synthesis, Excellent Water Stability, High $CO_2$ Selectivity and Fast $CO_2$ Transport. *J. Mater. Chem. A* **2016**, *4* (4), 1383–1389. DOI: 10.1039/c5ta09349j.

(15) Cousin-Saint-Remi, J.; Van der Perre, S.; Segato, T.; Delplancke, M.-P.; Goderis, S.; Terryn, H.; Baron, G.; Denayer, J. Highly Robust MOF Polymeric Beads with a Controllable Size for Molecular Separations. *ACS Appl. Mater. Interfaces* **2019**, *11*, 13694–13703. DOI: 10.1021/acsami.9b00521.

(16) Martín Morales, E.; Ruiz, K.; Llorente, A.; Biset-Peiró, M.; Guilera, J. Binder Effect on CeO2-Based Technical Catalyst Performance: Insights and Implications. *Catal. Today* **2024**, *429*. DOI: 10.1016/j.cattod.2023.114473.
(17) Wang R. rong; Zhang, J. liang; Liu, Y. ran; Zheng, A. yang; Liu, Z. jian; Liu, X. le; Li, Z. guo. Thermal Performance and Reduction Kinetic Analysis of Cold-Bonded Pellets with CO and H2 Mixtures. *Int. J. Miner. Metall. Mater.* **2018**, *25* (7), 752–761. DOI: 10.1007/s12613-018-1623-6.
